# Hypothalamic oxytocin neurons produce pro-anxiety effects through glutamatergic projections to the lateral hypothalamus

**DOI:** 10.1038/s41421-026-00925-1

**Published:** 2026-08-25

**Authors:** Xiao Cui, Qiuping Tong, Xuejin Ma, Xinyu Jiao, Ziling Wan, Qingjian Han, Xiangshan Yuan, Ruiqi Wu, Lei Xiao

**Affiliations:** 1 Shanghai Pudong Hospital, Fudan University Pudong Medical Center, State Key Laboratory of Brain Function and Disorders, MOE Frontiers Center for Brain Science, and the Institutes of Brain Science, Fudan University, Shanghai, China; 2 Department of Anatomy and Histoembryology, School of Basic Medical Sciences, Fudan University, Shanghai, China

**Keywords:** Mechanisms of disease, Cell signalling

Dear Editor,

Anxiety disorders represent the most prevalent class of psychiatric illnesses worldwide. Oxytocin (OXT), a well-known nonapeptide, has garnered attention for its therapeutic potential in affective disorders, including anxiety^1,2^. Direct infusion of OXT into emotion-related regions, such as central amygdala (CeA), anterior cingulate cortex (ACC), and dorsal raphe nucleus, has been shown to activate OXT receptors (OxtRs) and reduce anxiety and depression^3–5^. Endogenous OXT is primarily synthesized and released by neurons in the paraventricular nucleus of hypothalamus (PVN) and supraoptic nucleus (SON). Stimulating OXT axons within the CeA or ACC has also been shown to alleviate anxiety- and depression-like behaviors^3,4,6^. However, recent studies reported that chronic chemogenetic activation of PVN OXT neurons and chronic infusion of OXT promoted anxiety-like behaviors^7,8^, which contrasts with the anxiolytic effect of OXT. The contribution of OXT neuronal activity to anxiety remains to be elucidated. Neuronal functions critically depend on the released neuroactive molecules and circuit-specific projections. Some types of neurons are found to co-release multiple neurotransmitters or neuromodulators, which can even exert opposing functional effects^9,10^. PVN OXT neurons synthesize and co-release glutamate^11^. Although the functions of OXT released from PVN neurons have been extensively characterized^2,3,6,12^, the regions in which PVN OXT neurons release glutamate^13^, as well as the functional implications of this co-release, remain poorly understood.

We first investigated how acute optogenetic activation of PVN OXT neurons regulates anxiety-like behaviors. In mice, 20 Hz optogenetic stimulation of these neurons reduced locomotion speed and the time spent in the center region during the open-field test (OFT), and decreased the time spent in the open arms during the elevated plus maze test (EPM) (Fig. 1a, b; Supplementary Fig. S1a–c). This activation also elevated plasma corticosterone levels (Supplementary Fig. S1d). We next examined the frequency dependence of this effect. Stimulation at 40 Hz, but not 5 Hz, similarly reduced locomotion speed and the time spent in the center region during OFT, and both 20 Hz and 40 Hz stimulation led to a more pronounced reduction in the time spent in the center region than 5 Hz stimulation (Supplementary Fig. S1e, f). No significant effects were observed in mCherry-only control mice (Supplementary Fig. S1g). OXT is well known to promote social behaviors^2^; consistent with this, 20 Hz activation significantly increased social preference (Supplementary Fig. S1h). The anxiogenic effect of activating PVN OXT neurons was sex-invariant (Supplementary Fig. S1i, j). Together, these results demonstrate that acute optogenetic activation of PVN OXT neurons is sufficient to rapidly promote anxiety-like behaviors.

**Fig. 1:**
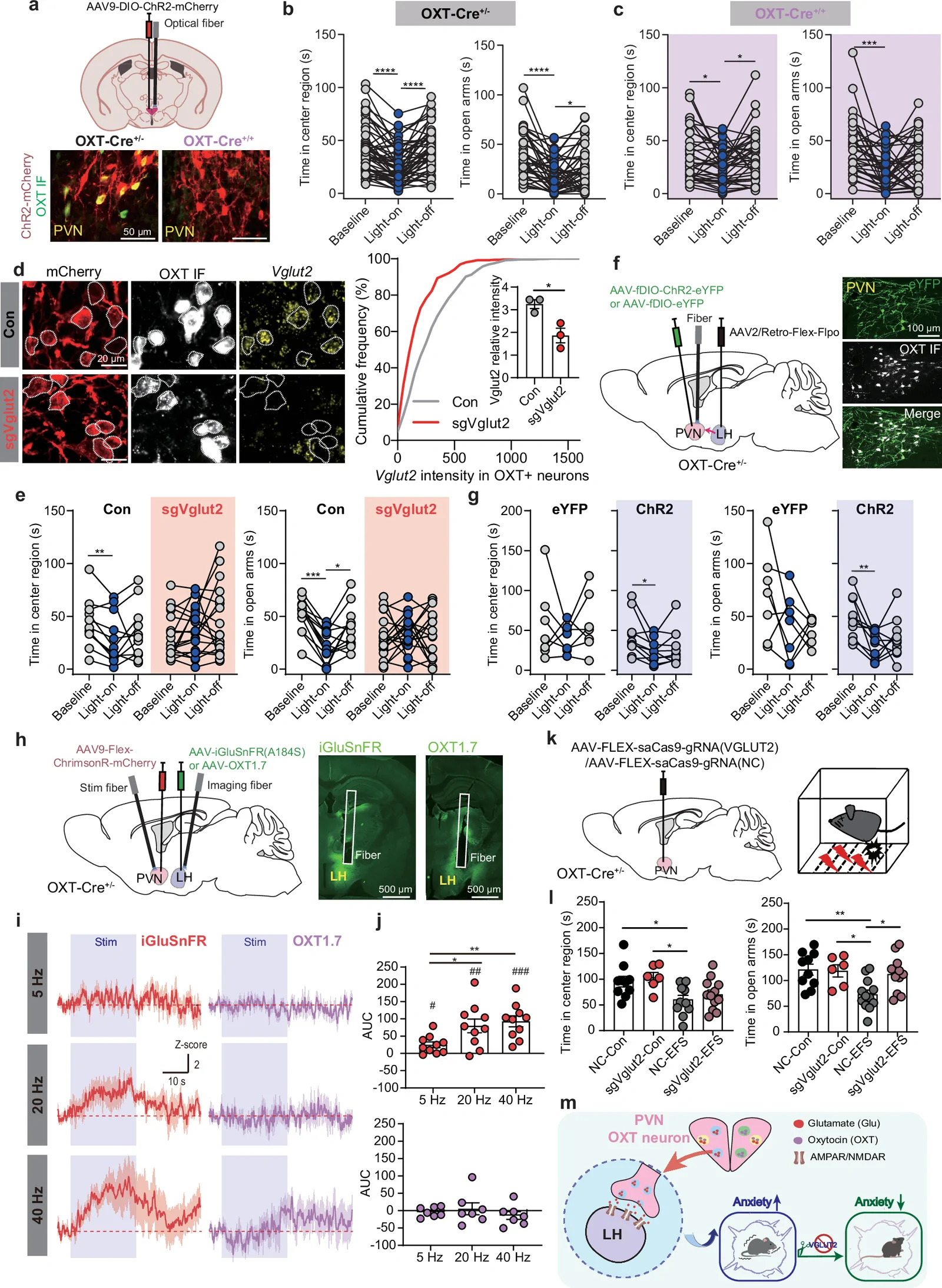
Optogenetic activation of PVN OXT neurons induces anxiety-like behaviors through glutamatergic projections to the LH. **a** Top: schematic of virus injection. Bottom: representative images showing ChR2-mCherry virus expression and OXT immunostaining from *Oxt-Cre*^*+/–*^ and *Oxt-Cre*^*+/+*^ mice. **b** Left: light-induced changes in the time spent in the center region during OFT. *n* = 54 mice. Right: light-induced changes in the time spent in the open arms during EPM. *n* = 41 mice. **c** Same as **b**, but for *Oxt-Cre*^*+/+*^ mice. *n* = 36 mice. **d** Left: representative images showing *Vglut2* expression in PVN OXT neurons (OXT IF (immunofluorescence)) in mice without (top, Con) and with (bottom, sgVglut2) *Vglut2* knockdown. Right: statistical results. *n* = 3 mice for each group. **e** Summary of the time spent in the center region during OFT (left) and the time spent in the open arms during EPM (right). *n* = 11 mice (Con group) and 18 mice (sgVglut2 group). **f** Left: strategy to activate LH-projecting PVN OXT neurons. Right: colocalization between eYFP^+^ neurons and OXT^+^ neurons. **g** Summaries of the time spent in the center region (left) and the time spent in the open arms (right) when stimulating LH-projecting PVN OXT neurons expressing only eYFP or ChR2-eYFP. *n* = 7 mice for eYFP and 10 mice for ChR2. **h** Left: schematic for monitoring glutamate and OXT release into the LH. Right: representative images showing iGluSnFR expression, OXT1.7 expression, and fiber implantation in the LH. **i** Average changes of iGluSnFR (left) and OXT1.7 (right) signals in the LH induced by activating OXT neurons. **j** Summary of light stimulation-induced glutamate (top) and OXT (bottom) signal changes in the LH. *n* = 10 mice for iGluSnFR group and 7 mice for OXT1.7 group. **k** Strategy to disrupt glutamate release from PVN OXT neurons and the EFS diagram. **l** Summary of the time spent in the center region and in the open arms. *n* = 10, 6, 12, and 12 mice for NC-Con, sgVglut2-Con, NC-EFS, and sgVglut2-EFS groups, respectively. **m** Schematic depiction of the main findings. *P < 0.05, **P < 0.01, ***P < 0.001, ****P < 0.0001, one-way ANOVA with Tukey post hoc tests or Friedman test with Dunn post hoc tests, or unpaired *t*-test. #*P* < 0.05, ##*P* < 0.01, ###*P* < 0.001, one-sample *t*-test.

To examine whether vesicle release from PVN OXT neurons is required for the anxiogenic signal, we co-expressed AAV-TetTox-mCherry and AAV-ChR2-eYFP in these neurons (Supplementary Fig. S2a). Blockade of vesicle release abolished the anxiogenic effects of optogenetic activation of PVN OXT neurons (Supplementary Fig. S2b, c), indicating the necessity of vesicular release. We next sought to determine whether OXT or co-released glutamate mediates this anxiogenic effect. OxtR antagonist L368899 can cross the blood–brain barrier. Following intraperitoneal injection of L368899 (5 mg/kg), optogenetic activation of PVN OXT neurons still promoted anxiety-like behaviors (Supplementary Fig. S2h–j), suggesting that OXT signaling may not be required for the anxiogenic effect. To further test this possibility, we utilized *Oxt-Cre*^*+/+*^ transgenic mice, in which Cre recombinase expression disrupts *Oxt* gene, thereby preventing OXT synthesis (Supplementary Fig. S2g). Activation of PVN Cre-positive neurons in these mice significantly reduced the time spent in the center region during OFT as well as the time spent in the open arms during EPM, with no sex difference (Fig. 1c; Supplementary Fig. S2d–f). Together, these results demonstrate that OXT release from PVN OXT neurons is not responsible for the anxiogenic effect.

To investigate whether glutamate release from PVN OXT neurons contributes to the anxiogenic effect, we used a Cre-dependent AAV vector expressing *Staphylococcus aureus* Cas9 (SaCas9) along with a single-guide RNA targeting *Vglut2* to disrupt glutamate release^10^. Selective expression of SaCas9 in PVN OXT neurons decreased *Vglut2* expression and glutamate release (Fig. 1d; Supplementary Fig. S3a–c). In the same treated animals, we additionally injected Cre-dependent ChR2-mCherry viruses into the PVN. Optogenetic activation of OXT neurons in these mice no longer reduced the time spent in the center region during OFT or the time spent in the open arms during EPM (Fig. 1e; Supplementary Fig. S3d–i). In contrast, control mice continued to exhibit anxiety-like behaviors upon stimulation (Fig. 1e; Supplementary Fig. S3g). Together, these results indicate that glutamate, rather than OXT, released from OXT neurons mediates the anxiogenic effect induced by their activation.

PVN OXT neurons have widespread projections to modulate diverse brain functions^2^. To identify the downstream regions through which OXT neurons promote anxiety-like behaviors, we first mapped their axonal projections by expressing AAV-DIO-Synaptophysin-eGFP in the PVN of *Oxt-Cre*^*+/–*^ mice (Supplementary Fig. S4a). eGFP-labeled axons were observed in several anxiety-related brain regions, such as CeA, nucleus accumbens (NAc), lateral septum (LS), lateral hypothalamic (LH), ventral tegmental area (VTA), and periaqueductal gray (PAG) (Supplementary Fig. S4b). Optogenetic activation of PVN OXT neurons increased cFos expression in all these downstream regions (Supplementary Fig. S4c–e), and functional magnetic resonance imaging (fMRI) consistently revealed elevated activity in the CeA, NAc, LS, LH, VTA, and PAG following PVN OXT neuron activation (Supplementary Fig. S5). To directly assess the behavioral contribution of each projection, we expressed ChR2 in PVN OXT neurons, implanted optical fibers above these downstream regions, and examined anxiety-like behaviors upon terminal stimulation. Optogenetic stimulation in the CeA significantly increased the time spent in the center region during OFT (Supplementary Fig. S6a), consistent with the previously reported anxiolytic effect of OXT in this region^4^. In contrast, terminal stimulation in NAc, LS, VTA, or PAG produced no significant changes in anxiety-like behaviors (Supplementary Fig. S6b, c, e, f). Notably, activation of OXT terminals in the LH robustly promoted anxiety-like behaviors, as evidenced by the decreased time spent in the center region and in the open arms (Supplementary Fig. S6d).

To further validate the role of LH-projecting PVN OXT neurons in the anxiogenic effect, we injected AAV2/Retro-Flex-Flpo viruses into the LH of *Oxt-Cre*^*+/–*^ mice and AAV-fDIO-ChR2-eYFP into the PVN (Fig. 1f). Selective activation of these LH-projecting OXT neurons recapitulated the anxiogenic phenotype (Fig. 1g), whereas selective activation of CeA-projecting OXT neurons had no effect (Supplementary Fig. S6g–i). We next examined whether LH-projecting PVN OXT neurons are necessary for inducing anxiety-like behaviors by selective ablation with caspase3 (Supplementary Fig. S7a). In eYFP-expressing control mice, optogenetic activation of PVN OXT neurons reduced the time spent in the center region and in the open arms. Importantly, these effects were abolished after selective ablation of LH-projecting PVN OXT neurons (Supplementary Fig. S7b–d). Collectively, these findings identify LH as a key downstream target through which PVN OXT neurons promote anxiety-like behaviors.

Given that knockdown of *Vglut2* in PVN OXT neurons abolished the anxiogenic effect (Fig. 1e), we hypothesized that these neurons primarily release glutamate into the LH. To test this, we first examined the colocalization of eGFP-labeled axons from PVN OXT neurons with OXT or Vglut2 in downstream brain regions. In the LH, we observed less colocalization of eGFP^+^ axons with OXT compared to that in the CeA (Supplementary Fig. S8a–c). In contrast, colocalization between eGFP^+^ axons and Vglut2 was more prominent in the LH (Supplementary Fig. S8d, e). Given that OXT is released from dense core vesicles (DCVs), we used electron microscopy to image synaptic boutons of PVN OXT neurons in the LH and CeA. Although both clear vesicles and DCVs were observed in the CeA, boutons in the LH appeared to mainly contain clear vesicles (Supplementary Fig. S9). These results reveal the region-specific neurochemical profiles of PVN OXT projections in the LH and CeA.

We next combined fiber photometry recording with optogenetic stimulation to separately monitor glutamate and OXT release into the LH and CeA upon activation of PVN OXT neurons (Fig. 1h; Supplementary Fig. S10). AAV9-Flex-ChrimsonR-mCherry was expressed in PVN OXT neurons, and AAV-iGluSnFR(A184S) or AAV-OXT1.7^12^ was injected into LH of different mice to monitor glutamate or OXT release, respectively (Fig. 1h). The same strategy was applied to detect glutamate and OXT release in the CeA (Supplementary Fig. S10a, b). In the LH, 5 Hz, 20 Hz, and 40 Hz red light stimulation evoked significant, frequency-dependent glutamate release, but only minimal OXT release (Fig. 1i, j). In contrast, in the CeA, activating PVN OXT neurons induced OXT release, but not glutamate release (Supplementary Fig. S10c–g). Together, these data demonstrate that PVN OXT neurons primarily release glutamate in the LH and OXT in the CeA.

Given that glutamate release from PVN OXT neurons contributes to anxiety-like behaviors, we hypothesized that disrupting this release would increase resilience to stress. To examine the role of glutamate release in an established anxiety mouse model, which was induced by five-day repeated electrical foot stimulation (EFS, 100 times, 0.5 mA, 5 s, and 5–60-s intervals), we disrupted the *Vglut2* gene in PVN OXT neurons of *Oxt-Cre*^*+/–*^ mice using AAV-FLEX-SaCas9-gRNA (VGLUT2) (Fig. 1k). The mice were divided into two groups: sgVglut2 (glutamate release disrupted) and NC (control). Within each group, mice either underwent EFS or received no stimulation (Con) (Supplementary Fig. S11a). NC-EFS mice exhibited pronounced anxiety-like behaviors compared to NC-Con mice, as shown by the decreased time spent in the center region and in the open arms (Fig. 1l; Supplementary Fig. S11b). Importantly, this EFS-induced anxiety phenotype was effectively rescued in sgVglut2-EFS mice (Fig. 1l). Consistent with these behavioral results, EFS significantly elevated *Vglut2* expression in PVN OXT neurons of NC-EFS mice compared with NC-Con mice (Supplementary Fig. S11c, d). We also investigated the effect of knockdown of PVN OXT expression by injecting AAV9-Cre-EGFP into the PVN of the *Oxt*^*flox/flox*^ mice (Supplementary Fig. S12a–e). Knockdown of PVN OXT expression did not alter mouse behaviors subjected to the EFS (Supplementary Fig. S12f, g). Together, these results indicate that glutamate release from PVN OXT neurons is involved in stress-induced anxiety.

In summary, our results demonstrate that glutamate released from PVN OXT neurons into LH promotes anxiety-like behaviors in a sex-conserved manner, and this glutamatergic pathway mediates chronic stress-induced anxiety (Fig. 1m). Our findings not only dissociate the opposing effects of glutamate and OXT release from PVN OXT neurons in anxiety modulation, but also reveal a previously unrecognized PVN → LH circuit contributing to the balanced regulation of anxiety-related behaviors in mice. While numerous studies reported the anxiolytic outcomes of exogenous administration of OXT into limbic regions such as amygdala^4^ and ACC^3^, clinical trials of intranasal OXT for anxiety and depression have yielded inconsistent results^1^. Some studies even observed that intranasal OXT increased anxiety under certain conditions^14^. Notably, intranasal OXT administration is reported to elevate endogenous OXT release^15^. Based on our findings, we propose that intranasal OXT not only elevates central OXT release through multiple potential routes but also may potentiate glutamate co-release from PVN OXT neurons, which will counteract the anxiolytic effects of OXT, though the precise mechanisms remain to be fully elucidated. Although the glutamate co-release from PVN OXT neurons has been reported in a fear-conditioning context in the CeA^13^, dissecting the distinct functional contributions of OXT and glutamate released from hypothalamic OXT neurons will be essential for advancing the translational application of OXT‑based therapies in brain disorders. In conclusion, our work establishes a direct causal link between the activity of PVN OXT neurons and anxiety levels, and the underlying mechanism extends beyond a purely peptidergic framework to incorporate the functional significance of glutamatergic co‑transmission. This co-transmission capacity enables a single neuronal population to exert complex and even opposing influences on emotional states, which is determined by the anatomical target of its projections. Future studies should therefore move past a monolithic view of OXT and instead focus on dissecting the specific circuit configurations, receptor localizations, and physiological contexts that determine whether its net outcome is anxiolytic or anxiogenic.

## Supplementary information

Supplementary information

## References

[1] De Cagna, F. et al. *Clin. Psychopharmacol. Neurosci.* **17**, 1–11 (2019). 30690935 10.9758/cpn.2019.17.1.1 PMC6361048

[2] Cui, X. & Xiao, L. *Neurosci. Bull.* **41**, 1267–1288 (2025). 40445489 10.1007/s12264-025-01424-1 PMC12229615

[3] Li, X. H. et al. *Cell Rep.* **36**, 109411 (2021). 34289348 10.1016/j.celrep.2021.109411

[4] Li, Y. et al. *CNS Neurosci. Ther.* **29**, 3493–3506 (2023). 37248645 10.1111/cns.14282 PMC10580334

[5] Yoshida, M., Takayanagi, Y., Inoue, K., Kimura, T., Young, L. J. & Onaka, T. *J. Neurosci*. **29**, 2259–2271 (2009). 19228979 10.1523/JNEUROSCI.5593-08.2009 PMC6666325

[6] Xia, J. et al. *Proc. Natl. Acad. Sci.**USA* **122**, e2503675122 (2025).

[7] Winter, J. et al. *Mol. Psychiatry* **28**, 4742–4755 (2023). 34035479 10.1038/s41380-021-01141-x PMC10914602

[8] Tan, H. et al. *Neurosci. Bull.* **42**, 945–960 (2026). 40847250 10.1007/s12264-025-01475-4 PMC13158320

[9] Soden, M. E., Yee, J. X. & Zweifel, L. S. *Nature* **619**, 332–337 (2023). 37380765 10.1038/s41586-023-06246-7 PMC10947507

[10] Warlow, S. M. et al. *Neuron* **112**, 488–499.e5 (2024). 38086374 10.1016/j.neuron.2023.11.002 PMC10922836

[11] Osakada, T. et al. *Nature* **626**, 347–356 (2024). 38267576 10.1038/s41586-023-06958-w PMC11102773

[12] Xu, H. et al. *Sci. Adv.* **12**, eaef8400 (2026). 42284412 10.1126/sciadv.aef8400 PMC13262635

[13] Hasan, M. T. et al. *Neuron* **103**, 133–146.e8 (2019). 31104950 10.1016/j.neuron.2019.04.029

[14] Eckstein, M., Scheele, D., Weber, K., Stoffel-Wagner, B., Maier, W. & Hurlemann, R. *Hum. Brain Mapp.* **35**, 4741–4750 (2014). 24659430 10.1002/hbm.22508 PMC6869318

[15] Quintana, D. S., Lischke, A., Grace, S., Scheele, D., Ma, Y. & Becker, B. *Mol. Psychiatry* **26**, 80–91 (2021). 32807845 10.1038/s41380-020-00864-7 PMC7815514

