# Supplementary material for: Hypothalamic oxytocin neurons produce pro-anxiety effects through glutamatergic projections to the lateral hypothalamus
Source: Cell Discov. 2026 Aug 25;12:63. doi: 10.1038/s41421-026-00925-1 (PMC13503807; doi:10.1038/s41421-026-00925-1)
Supplement: Supplementary file 1 [file 41421_2026_925_MOESM1_ESM.pdf]

Supplementary Information for

Hypothalamic oxytocin neurons produce pro-anxiety effects through glutamatergic projections to the lateral hypothalamus

Xiao Cui[1,#], Qiuping Tong[1,#], Xuejin Ma[1,#], Xinyu Jiao[1], Ziling Wan[1], Qingjian Han[1], Xiangshan Yuan[2,*], Ruiqi Wu[1,*], Lei Xiao[1,*]

[1] Shanghai Pudong Hospital, Fudan University Pudong Medical Center, State Key Laboratory of Brain Function and Disorders, MOE Frontiers Center for Brain Science, and the Institutes of Brain Science, Fudan University, Shanghai, 200032, China

[2] Department of Anatomy and Histoembryology, School of Basic Medical Sciences, Fudan University, Shanghai, 200032, China

[#] X.C., Q.T., and X.M. contributed equally to this work.

*Correspondence: Xiangshan Yuan (yuanxiangshan1999@163.com), Ruiqi Wu (ruiqiwu@fudan.edu.cn), and Lei Xiao (leixiao@fudan.edu.cn)

This PDF file includes:

Materials and Methods

Supplementary information, Figs. S1-S12

Materials and Methods

Mouse strains and genotyping. All animal procedures were approved by the Fudan University Animal Care and Use Committee (No. 20230301-139). Adult mice (6-12 weeks) were group-housed on a 12:12 light-dark cycle (8 am light-ON and 8 pm light-OFF) with *ad libitum* access to standard chow diet and water in the room that was controlled for temperature and humidity. Mouse lines used in this study including: C57BL/6 mice were obtained from Shanghai Model Organisms Center; B6.129S-Oxt$^{1.1(cre)Dolsn}$/J mice (OXT-Cre, #024234, Jackson Laboratory), which express Cre recombinase under the control of oxytocin promoter [1], were used to target PVN oxytocin neurons; OXT$^{flox/flox}$ mice (C57BL/6Smoc-Oxt$^{em1(flox)Smoc}$, NM-CKO-2115124) were used to conditional knock down OXT expression [2]. Mouse genotyping was conducted following standard procedures on the Jackson Lab website. Both males and females were used in this study.

***In vivo* stereotaxic intracranial injection.** Mice aged 6-8 weeks were anesthetized with 0.5%-2% isoflurane and placed in a stereotaxic apparatus (E07370-005, RWD). For virus injection, recombinant AAVs were delivered into brain regions by a microsyringe pump controller (NanoJect III, Drummond Scientific Company) at a volume of 50-400 nL with a flow rate of 1-2 nL/s. Injection pipette was left at the injection site for at least 10 min after viral delivery to assure adequate viral delivery. The following coordinates were used: PVN (0.2 mm posterior to bregma, 0.3 mm lateral to the midline, 4.6 and 4.9 mm below the pia), LH (0.6 mm posterior to bregma, 1.1 mm lateral to the midline, 5.2 mm below the pia), CeA (1.2 mm posterior to bregma, 3.0 mm lateral to the midline, 4.4 mm below the pia). Coordinates were slightly adjusted based on mouse age and brain size.

The following AAV viruses were used in this study: AAV9-hSyn-DIO-mCherry (4.2×10^{12} vector genomes/mL, Taitool), AAV9-hEF1a-DIO-hChR2(H134R)-mCherry (3.4×10^{12} vector genomes/mL,

Taitool), AAV2/9-hEF1α-DIO-mCherry-P2A-TetTox-WPRE-pA (1.4 × 10^{13} vector genomes/mL, Taitool), rAAV-EF1a-DIO-hChR2(H134R)-eYFP-WPRE-hGH (1.4 × 10^{13} vector genomes/mL, BrainVTA), AAV2/9-hEFla-DIO-Synaptophysin-eGFP (1.1 × 10^{13} vector genomes/mL, Taitool), AAV2-Retro-CAG-Flex-Flpo (1.66 × 10^{13} vector genomes/mL, Taitool), AAV9-hEF1a-fDIO-hChR2(H134R)-EYFP (1.65 × 10^{12} vector genomes/mL, Taitool), AAV2/9-hEF1a-fDIO-EYFP (1.16 × 10^{12} vector genomes/mL, Taitool), AAV2/9-hEF1a-fDIO-taCaspase3 (1.53 × 10^{13} vector genomes/mL, Taitool), AAV2/9-hsyn-Flex-ChrimsonR-mCherry (2.06 × 10^{12} vector genomes/mL, Taitool), AAV2/9-hSyn-OXT1.7 (5.88 × 10^{12} vector genomes/mL, BrainVTA) [2], AAV-hsyn-OXTmut (6.16 × 10^{12} vector genomes/mL, BrainVTA), AAV2/9-hSyn-SF-iGluSnFR(A184S) (1.42 × 10^{13} vector genomes/mL, Taitool) [3], AAV2/9-hSyn-Cre-eGFP-WPRE-pA (1.87 × 10^{13} vector genomes/mL, Taitool).

A single adeno-associated virus (AAV) vector was constructed for Cre-dependent expression of *Staphylococcus aureus* Cas9 (SaCas9) together with a single-guide RNA (sgRNA, TATGCTGATCCCATCTGCAGC) targeted to the gene encoding *Vglut2* (*Slc17a6*). The reconstructed vectors were packaged in serotype adeno-associated virus 9 (AAV9). The reconstruction and packaging were performed by the Taitool (China), yielding the following two preparations: AAV2/9-hSyn-FLEX-saCas9-HA-SV40pA-hU6-gRNA (Vglut2) (2.02 × 10^{12} vector genomes/mL, Taitool), AAV2/9-hSyn-FLEX-saCas9-HA-SV40pA-hU6-gRNA (NC) (3.33 × 10^{12} vector genomes/mL, Taitool). The sgRNA sequence was designed as previously reported [4].

Optical fiber implantation. For *in vivo* photostimulation experiments, an optical fiber (200 μm, 0.37 NA, Newdoon) was implanted into targeted brain regions, including PVN (0.2 mm posterior to bregma, 0.3 mm lateral to the midline, 4.4 mm below the pia), LH (0.6 mm posterior to bregma, 1.2

mm lateral to the midline, 4.7 mm below the pia), CeA (1.2 mm posterior to bregma, 3.0 mm lateral to the midline, 4.0 mm below the pia), NAc (1.6 mm anterior to bregma, 1.0 mm lateral to the midline, 4.0 mm below the pia), LS (0.6 mm anterior to bregma, 0.3 mm lateral to the midline, 2.7 mm below the pia), VTA (2.9 mm posterior to bregma, 0.3 mm lateral to the midline, 4.2 mm below the pia), and PAG (3.6 mm posterior to bregma, 0.3 mm lateral to the midline, 2.9 mm below the pia), using stereotaxic apparatus at least 3 weeks after viral injection surgery.

Optical fibers were fixed to the skull with dental cement. After at least one week of the fiber implantation experiment, the mice underwent behavioral tests. Following all behavioral experiments, brains were sectioned to verify fiber placement and injection sites. Animals with incorrect placements were excluded from analysis.

Acute slice preparation and electrophysiological recording. Mouse acute brain slices were prepared as previously described [5–7]. Briefly, mice were anesthetized with isoflurane and transcardially perfused with the ice-cold, oxygenated artificial cerebrospinal fluid (ACSF) containing (in mM): 213 surcose, 2.5 KCl, 26 $NaHCO_3$, 1.25 NaH_2PO_4, 2 $CaCl_2$, 2 $MgCl_2$, and 10 glucose (Osmolarity ~310 mOsm/L). The brain was quickly removed and immersed in ice-cold, oxygenated (95% O_2 / 5% CO_2) ACSF in a slicing chamber. Coronal slices (~ 250 μm thick) were cut using a vibrating microtome (Vibratome 1000 PLUS) and incubated in the oxygenated ACSF containing (in mM): 127 NaCl, 2.5 KCl, 25 $NaHCO_3$, 1.25 NaH_2PO_4, 2 $CaCl_2$, 1 $MgCl_2$, and 25 glucose (Osmolarity ~310 mOsm/L), and maintained at 34°C for ~30 min before recording.

For recording, slices were transferred to a recording chamber perfused with oxygenated ACSF (1.5-2 mL/min) and maintained at ~ 30°C during recording with a feedback-controlled in-line heater (TC-324B; Warner Instruments, Hamden, CT). Fluorescently labeled PVN oxytocin neurons were

identified using infrared differential interference contrast microscopy. Whole-cell - attached recordings of action potentials were performed using an internal solution containing (in mM): 135 K-gluconate, 4 KCl, 20 Hepes, 10 Na-phosphocreatine, 2 Mg adenosine 5'-triphosphate, and 0.2 Na_2 guanosine 5'- triphosphate (with pH 7.2 – 7.3, and osmolarity ~295 mOsm/L). For optogenetic activation, 10-ms-long light pulses (470 nm, 20 Hz for 5 s, and 0.2 to 2.5 mW/mm^2) were delivered at the recording site using whole-field illumination through a 40× water immersion objective (Nikon, Tokyo, Japan) with a PE300 CoolLED illumination system (CoolLEDLtd., Andover, UK). Neuronal spontaneous activities were recorded under the current-clamp mode (Ra < 30 megohm), and data were filtered at 4 kHz and sampled at 10 kHz using an Integrated Patch-Clamp Amplifier (Sutter) with Igor Pro (Wave Metrics, Inc.) data acquisition software.

Tissue processing, immunohistochemistry, and imaging. Mice were deeply anesthetized with isoflurane and transcardially perfused with 4% paraformaldehyde (PFA) in 0.1 M phosphate-buffered saline (PBS). Brains were dissected and post-fixed in 4% PFA at 4°C for 1-2 days, and washed in PBS. Coronal sections (50-60 μm) were cut on a vibratome (VT1200, Leica, Germany). Every third section was chosen for immunohistochemistry. Sections were permeabilized in PBS containing 0.2% Triton X-100 for 1 h at room temperature (RT), then blocked in PBS containing 0.05% Triton X-100 and 10% bovine serum albumin (BSA) for 1 h at RT, followed by PBS washes. Sections were incubated overnight at 4 °C with primary antibodies diluted in PBS containing 0.2% Triton X-100: rabbit anti-OXT (1:1000; T-4084, Peninsula Laboratories), rabbit anti-vasopressin (1:1000; 20069, Immunostar), guinea pig anti-c-Fos (1:5000; 226308, Synaptic Systems), and Rat anti-Vglut2 (1:1000; 135402, Synaptic Systems). After washing three times with PBS, sections were incubated for 2 h at RT with secondary antibodies diluted in PBS: goat anti-rabbit IgG conjugated to Alexa Fluor 488 or 647 (1:1000; Thermo Fisher) and goat anti-guinea pig IgG (1:1000; Vector Laboratories). For

c-Fos detection, sections were subsequently incubated for 2 h at RT with Alexa Fluor 647-conjugated tertiary antibody (1:1000; Thermo Fisher). Following final PBS washes, sections were mounted on slides, air-dried, and coverslipped with a glycerol-based mounting medium (glycerol:TBS, 3:1) containing Hoechst 33342 (1:1000; Thermo Fisher). Sections were imaged with an Olympus VS120 slide-scanning microscope using a 10× objective. High-resolution confocal images were acquired with a Nikon A1 confocal laser scanning microscope with a 20× or 60× objective. Images were analyzed in ImageJ (FIJI).

Electron microscopy. Samples were prepared as previously described [8]. Under deep anesthesia with isoflurane, OXT-Cre mice injected with AAV2/9-hEFla-DIO-Synaptophysin-eGFP in PVN were briefly perfused transcardially with 6–8 mL saline, followed by 100 mL ice-cold fixative containing 4% PFA, 0.5% glutaraldehyde, and 15% saturated picric acid in 0.1 M PB (pH 7.4). Immediately after perfusion-fixation, brains were removed and postfixed in 4% PFA for an additional 2 h at 4°C. Brain samples containing the LH and CeA were cut into coronal sections (50 μm thick) with a vibratome (VT1000S, Leica) and collected in 0.05 M PB for eGFP staining. The sections were placed in 0.05 M PB (pH 7.4) containing 25% (w/v) sucrose and 10% (v/v) glycerol for 1 h and then freeze-thawed with liquid nitrogen to enhance antibody penetration. The sections were then incubated in chicken anti-eGFP antibody (1:2000, GFP-1020, Aves Labs) in 0.05 M PB containing 5% normal donkey serum for 30–36 h at 4°C. Next, the sections were incubated with donkey anti-chicken biotinylated IgG (1:750, Jackson ImmunoResearch) for 3 h at RT, followed by incubation in ABC complex for 3 h at RT. After eGFP immunoractivity was visualized with DAB (SK-4100, Vector Laboratories) following the same procedure used for light microscopy.

The eGFP-labeled sections were osmicated with 2% OsO_4, dehydrated in a graded series of ethanol,

and embedded flat in Epon 12 (Ted Pella, Redding, USA) using embedding capsules (TAAB, Berks, UK). The sections embedded in Epon were observed under a light microscope, and areas of the LH and CeA were sampled. Ultrathin sections were cut at a thickness of 70 nm using an ultramicrotome, stained with uranyl acetate and lead citrate, and then examined in a Talos L120C transmission electron microscope (Thermo, USA).

Behavioral tests

All behavioral tests were conducted between 10:00 am – 8:00 pm. Mice were habituated to the testing room for at least 1 h prior to each experiment and were randomly assigned to be control or experimental groups. If not specifically stated, behavioral tests were performed with light-on. Behavioral apparatuses were cleaned with 75% ethanol to eliminate residual odor clues between trials. Only one behavioral paradigm was performed per session for pharmacological or optogenetic manipulations, with an interval of at least 5 days between different pharmacological tests.

For the optogenetic manipulation, 10-ms laser pulses of 473 nm blue light (~10 mW at the fiber tip) were delivered in 5s-on-5s-off cycles at 5 Hz, 20 Hz, or 40 Hz using a solid-state laser, and stimulation patterns were controlled by the DigiStim (Thinker Tech Nanjing Bioscience Inc). One day before the first behavioral test, mice were connected to the patch cord and habituated to the optogenetic setup for at least 15 min. Behavioral trajectories were tracked using Toxtrac software (https://toxtrac.sourceforge.io) and analyzed with custom MATLAB scripts [7].

Open field test. Mice were allowed to freely explore an open-field arena (42 cm × 42 cm × 50 cm) while their movement was tracked in real time using an infrared video camera for a total of 10 min or 20 min. The time spent in the center zone (defined as the central 50% of the total area) and the

locomotion speed were recorded for analysis. In optogenetic experiments, the session was divided into four consecutive 5-min phases: Habituation stage, Baseline, Light-on, and Light-off. The same arena was cleaned with 75% ethanol between trials to remove olfactory cues.

Elevated plus maze (EPM) test. The maze consisted of a center platform (6 cm × 6 cm), two open arms (35 cm × 6 cm), and two closed arms (35 cm × 6 cm × 19 cm), elevated 73 cm above the floor. Each mouse was placed in the center platform facing one open arm and allowed to explore freely for 5 min or 10 min while being recorded with an infrared video camera. The number of entries into and time spent in the open arms were quantified. For the optogenetic experiment, the 10-min session was divided into four consecutive 2.5-min phases: Habituation stage, Baseline, Light-on, and Light-off. Any animal that fell off the maze was excluded from analysis. The apparatus was cleaned with 75% ethanol between trials.

Three-chamber social preference test. The apparatus consisted of a clear plexiglass box (60 cm × 40 cm × 44 cm) divided into three equal chambers (each 20 cm × 40 cm × 44 cm) with removable doors in the central chamber. On day 1, mice were allowed to freely explore and habituate to the entire empty arena for 15 min, with two empty transparent enclosures placed in opposite corners of the two side chambers. On day 2, each mouse was first placed in the central chamber for a 10-min free-exploration session. Subsequently, a novel, unfamiliar mouse (same sex and similar age) was placed inside one enclosure, while an inanimate object (plastic mouse toy) was placed in the other enclosure; the test mouse was then allowed to explore for an additional 10 min. All sessions were recorded with an infrared video camera. Social preference was calculated as: (time in social zone) / (time in social zone + time in non-social zone). In optogenetic experiments, laser stimulation was delivered throughout the second 10-min session (social vs. object phase). The apparatus was

cleaned with 75% ethanol between trials.

Quantitative fluorescence in situ hybridization (FISH). The FISH experiment was conducted as previously described [7,9,10]. Brain sections containing the PVN from OXT-Cre mice were processed for FISH according to the manufacturer's protocol (RNAscope® Fluorescent Multiplex Assay v2; Advanced Cell Diagnostics, Newark, CA). Following FISH, selected sections were further processed for immunohistochemistry using the following primary antibodies (incubated for 24 h at 4 °C): rabbit anti-OXT (1:800; T-4084, Peninsula Laboratories), goat anti-mCherry (AB0081-200, Sicgen), and chicken anti-GFP (1:800; AB13970, Abcam). After washing, sections were incubated with the corresponding secondary antibodies: goat anti-rabbit Alexa Fluor 647 (1:800; Thermo Fisher), donkey anti-goat Alexa Fluor 555 (1:800; Thermo Fisher), and goat anti-chicken Alexa Fluor 488 (1:800; Life Technologies). Sections were coverslipped with ProLong Gold Antifade Mountant containing DAPI (Molecular Probes). *Slc17a6* (*Vglut2*, C1, 318171) was used in this study. Control reactions included probe omission or negative control probes for each experiment. Sections were imaged with a Nikon A1 confocal laser scanning microscope with a 20× water objective (1 μm z-step). All channels were thresholded to remove background noise. Cellular regions of interest (ROIs) were defined based on mCherry or eGFP immunofluorescence to localize cell bodies. Images were processed with ImageJ (FIJI, NIH). All quantification was performed by an experimenter blinded to the experimental conditions.

Enzyme-linked immunosorbent assay for detecting serum corticosterone (CORT-ELISA). Orbital blood was collected and kept at room temperature for 15 min, followed by incubation on ice for 30 min. Samples were centrifuged at 3000 rpm for 15 min at 4 °C, and the supernatant (serum) was collected and kept at -80 °C before use. Serum was diluted according to the manufacturer's

protocol, and corticosterone concentration was measured using a commercial ELISA kit (JL11918, Jianglai Biotech, Shanghai, China). A standard curve was generated on the same plate using serial dilutions of corticosterone; all standard curves exhibited an $R^2 > 0.9$. Sample values were normalized based on the standard curve.

RNA Extraction and Reverse Transcription-Quantitative PCR (RT-qPCR). Mice were deeply anesthetized with isoflurane and transcardially perfused with ice-cold PBS. Coronal sections containing PVN region were removed on ice using a mouse brain matrix (RWD) allowing 1 mm sections, and the region was dissected precisely under the microscope and rapidly frozen in liquid nitrogen. Tissues were homogenized, and total RNA was isolated using Trizol reagent (TianGen, China) according to the manufacturer's instructions and kept at -80 °C before use. Aliquots containing 800 ng RNA from each sample were reverse transcribed into cDNA using HiScript III RT SuperMix (Vazyme, China). The ChamQ Universal SYBR qPCR Master Mix (Vazyme, China) was used for real-time PCR amplification. Real-time PCR was performed on QuantStudio 3 RealTime PCR Systems (Thermo Fisher Scientific), and quantification was performed using QuantStudio Design & Analysis Software (Thermo Fisher Scientific). Murine β-actin was used as a reference to normalize the targeted gene expression levels. The following primers were used:

5'-CAGGATGCAGAAGGAGATTAC-3' (forward)

5'-AACGCAGCTCAGTAACAGTC-3' (reverse) for actin;

5'-CTGTGCTGGACCTGGATATGCG-3' (forward)

5'-AGCTCGTCCGCGCAGCAGATG-3' (reverse) for OXT;

5'-CCTATGCTGGAGCAGTCATTGC-3' (forward)

5'-GGCTCTC ATAAGACACCAGAAGC-3' (reverse) for Vglut2;

Fiber photometry recording. Fiber photometry recording to monitor glutamate or OXT release from PVN OXT neurons in freely behaving testing. One day prior to behavioral testing, animals were connected to a patch cord and habituated to the tethering setup in an open-field arena (42 cm × 42 cm × 50 cm) for at least 15 min. During recording, mice were tethered via a 200-μm, 0.37-NA optical fiber patch cord connected to a commercial fiber-photometry system (Thinker Tech Nanjing Biotech). For optogenetic stimulation of PVN OXT neurons, 10-ms pulses of 647-nm red light (~13 mW at the fiber tip) were delivered in 20-s-on/60-s-off cycles at 5, 20, or 40 Hz. Fluorescence signals were acquired simultaneously at 405 nm (isosbestic reference) and 470 nm (calcium-dependent or sensor-specific) channels. Recorded signals were normalized as Z-scores to quantify stimulus-locked release dynamics of glutamate or OXT. For each animal, the area under the curve (AUC) and peak amplitude of the signal during the stimulation window (0–20 s) were extracted and averaged per group using custom MATLAB scripts. After experiments, fiber placement was verified histologically.

Functional magnetic resonance imaging (fMRI). To assess brain-wide responses to activation of PVN OXT neurons, we expressed ChR2 in PVN OXT neurons and performed fMRI during optogenetic stimulation. Data were acquired on a 9.4 T animal scanner (uMR 9.4 T, United Imaging Life Science Instrument, China) using a custom single-loop surface coil. The detailed procedures were similar to those in our previous studies on mouse fMRI [11]. Briefly, mice were initially anesthetized with isoflurane (4% in 1:4 O_2/air) and received an intraperitoneal bolus of dexmedetomidine (0.016 mg/kg). After placement on the scanner bed, light anesthesia was maintained with continuous isoflurane (0.6% in 1:4 O_2/air) and dexmedetomidine infusion

(0.096 mg/kg/h, i.p.). Body temperature was monitored via a rectal probe and kept at 37.5 ± 0.5 °C throughout the experiment. For optogenetic stimulation, 473 nm laser pulses (20 Hz, ~10 mW at the fiber tip) were delivered for 20 s (Changchun New Industries).

Functional images were acquired using a gradient-echo echo-planar imaging (GE-EPI) sequence with the following parameters: TR = 2000 ms, TE = 17 ms, flip angle = 60°, in-plane resolution = 0.20 × 0.20 mm², 21 slices, slice thickness = 0.35 mm, 150 repetitions. High-resolution anatomical images were obtained with a fast spin-echo (FSE) T2-weighted sequence: TR = 3000 ms, TE = 37.56 ms, in-plane resolution = 0.20 × 0.20 mm², 21 slices, slice thickness = 0.35 mm, 8 averages.

fMRI preprocessing and analysis were performed using the Rodent Automated Bold Improvement of EPI Sequences (RABIES) pipeline (https://github.com/CoBrALab/RABIES) [12], Analysis of Functional NeuroImages (AFNI; NIH, USA), and custom MATLAB scripts. Preprocessing included slice-timing correction, motion realignment, nonlinear registration to a study-specific anatomical template, band-pass filtering (0.001–0.2 Hz), and spatial smoothing (0.6 mm FWHM Gaussian kernel). Statistical activation maps were generated using a general linear model with a double-gamma hemodynamic response regressor. To quantify responses in regions identified by c-Fos mapping, mean BOLD time courses were extracted from the NAc, LS, LH, CeA, VTA, and PAG. Signals were normalized to a 20-s pre-stimulus baseline, and percent BOLD change was calculated. Region-of-interest (ROI) masks were defined as the overlap between manually annotated anatomical ROIs and the group-specific activation map. The mean signal and area under the curve (AUC) were computed over the 40 s post-stimulus period.

Electric foot shock. Mice aged 8 to 10 weeks were exposed to electric foot shocks in fear conditioning chambers (28 cm x 29 cm x 28 cm) [6]. A total of 100 intermittent inescapable electric foot shocks (0.5 mA, 5 s, and 5- to 60-s intervals) were delivered once daily for five consecutive days. Mice in the control group were exposed to the fear conditioning chambers for an equivalent amount of time without an electric foot shock. The fear conditioning chambers were thoroughly cleaned with 75% ethanol between tests to avoid interference from the previous subject. Five days after the electric foot shock, behavioral tests were performed to evaluate anxiety-like behaviors in the mice.

Quantification and Statistical Analysis. No statistical method was used to pre-determine sample sizes, and they were chosen based on established practice in related studies. Behavioral and electrophysiological data were analyzed using MATLAB R2022 (MathWorks), pCLAMP 10 (Molecular Devices), or GraphPad Prism 9.5 (GraphPad). Images were processed with ImageJ (FIJI, NIH). Whenever possible, analyses were performed blind to experimental conditions. Group data were expressed as mean ± SEM. Normality was assessed with the Shapiro-Wilk test. For normally distributed data with homogeneous variance (tested by F-test), comparisons between two groups were made using two-tailed unpaired or paired t-tests; multiple comparisons were performed with one-way ANOVA followed by Tukey's post-hoc test. If variances were unequal, a t-test with Welch's correction was applied. For non-normally distributed data, the Mann-Whitney test (unpaired), Wilcoxon signed-rank test (paired), or Wilcoxon matched-pairs signed-rank test was used for two-group comparisons; multiple comparisons were analyzed with the Friedman test followed by Dunn's post-hoc test. A p-value < 0.05 was considered statistically significant.

References

1. Shah BP, Vong L, Olson DP, *et al.* MC4R-expressing glutamatergic neurons in the paraventricular

hypothalamus regulate feeding and are synaptically connected to the parabrachial nucleus. *Proceedings of the National Academy of Sciences* 2014; **111**:13193–13198.

2. Liu C, Wang X, Yao J, *et al.* Distinct projections of PVN oxytocin neurons regulate the divergent defensive behaviors in response to social threat. *Science Advances* 2026; **12**:eaeb0400.

3. Qian T, Wang H, Wang P, *et al.* A genetically encoded sensor measures temporal oxytocin release from different neuronal compartments. *Nature Biotechnology* 2023; **41**:944–957.

4. Marvin JS, Scholl B, Wilson DE, *et al.* Stability, affinity, and chromatic variants of the glutamate sensor iGluSnFR. *Nat Methods* 2018; **15**:936–939.

5. Warlow SM, Singhal SM, Hollon NG, *et al.* Mesoaccumbal glutamate neurons drive reward via glutamate release but aversion via dopamine co-release. *Neuron* 2023:S0896627323008462.

6. Chen S, Xu H, Dong S, Xiao L. Morpho-electric properties and diversity of oxytocin neurons in paraventricular nucleus of hypothalamus in female and male mice. *The Journal of neuroscience : the official journal of the Society for Neuroscience* 2022; **42**:2885–2904.

7. Zhang J, Xie C, Xu P, Tong Q, Xiao L, Zhong J. Projections from subfornical organ to bed nucleus of the stria terminalis modulate inflammation-induced anxiety-like behaviors in mice. *Science Advances* 2024; **10**:eadp9413.

8. Tong Q, Cui X, Xu H, *et al.* D1 receptor-expressing neurons in ventral tegmental area alleviate mouse anxiety-like behaviors via glutamatergic projection to lateral septum. *Molecular Psychiatry* 2023; **28**:625–638.

9. Yuan X, Xiang Z, Jiang J, *et al.* Leptin receptor neurons in the ventral premammillary nucleus modulate emotion-induced insomnia. *Cell Discov* 2024; **10**:59.

10. Xiao L, Priest MF, Kozorovitskiy Y. Oxytocin functions as a spatiotemporal filter for excitatory synaptic inputs to VTA dopamine neurons. *eLife* 2018; **7**:e33892.

11. Wang Y, Xu H, Chen S, *et al.* Oxytocin Protects Nigrostriatal Dopamine Signal via Activating GABAergic Circuit in the MPTP-Induced Parkinson's Disease Model. *Advanced Science* 2024; **11**:e2310244.

12. Zhang C. TMC6 functions as a GPCR-like receptor to sense noxious heat via Gαq signaling. *Cell Discovery* 2024; **10**:66.

13. Desrosiers-Grégoire G, Devenyi GA, Grandjean J, Chakravarty MM. A standardized image processing and data quality platform for rodent fMRI. *Nat Commun* 2024; **15**:6708.

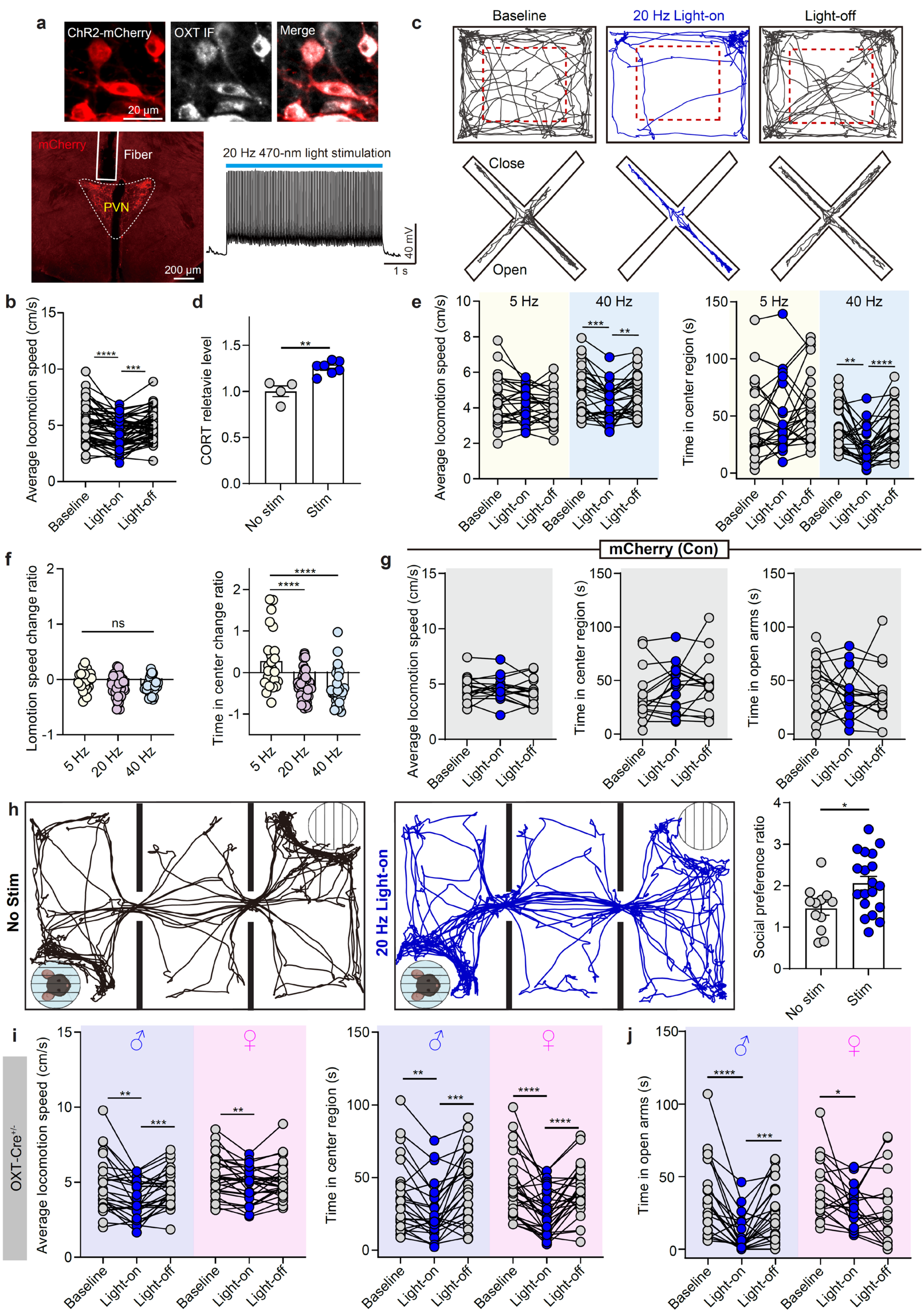

Supplementary Figure S1. Optogenetic activation of PVN OXT neurons induces anxiety-like

behaviors. (**a**) Top: Examples showing ChR2-mCherry virus expression and OXT immunostaining in PVN OXT neurons. Bottom: example of virus injection and optic fiber implantation and light-evoked action potentials in one mCherry+ neuron. (**b**) Summaries about average locomotion speed in the OFT. n = 54 mice. (**c**) Top: Examples showing the movement trajectories of one mouse with ChR2-mCherry expression in PVN OXT neurons in the OFT before, during, and after 20 Hz light stimulation. Bottom: Examples showing the trajectories of one mouse with ChR2-mCherry expression in PVN OXT neurons in the EPM. (**d**) Plasma corticosterone (CORT) levels in ChR2-mCherry expression mice with (Stim) and without (No stim) light stimulation. n = 4 and 7 mice for the No stim and Stim groups, respectively. (**e**) Summaries about average locomotion speed (Left) and time spent in the center region (Right) in the OFT. n = 25 and 27 mice for 5 Hz and 40 Hz light stimulation, respectively. (**f**) Summaries of the light-induced changes ratio in average locomotion speed and time spent in the center region across frequencies in the OFT test. n = 25, 52 and 27 mice for 5 Hz, 20 Hz, and 40 Hz light stimulation, respectively. (**g**) Summaries of the light stimulation-induced behavioral alterations in control mice with only mCherry expression. n = 16 mice. (**h**) Left: The example trajectories of one mouse in the social preference test with and without light stimulation. Right: Summary of social preference ratio in mice with and without light stimulation. (**i**) Summaries about locomotion speed (Left) and time spent in the center region (Right) in the OFT from OXT-Cre$^{+/-}$ male and female mice. n = 22 males and 19 females. (**j**) Same as (**i**), but the EPM test. n = 12 and 19 mice for the No stim and Stim groups, respectively. * $p < 0.05$, ** $p < 0.01$, *** $p < 0.001$, **** $p < 0.0001$, one-way ANOVA with Tukey *post hoc* tests or unpaired t-tests.

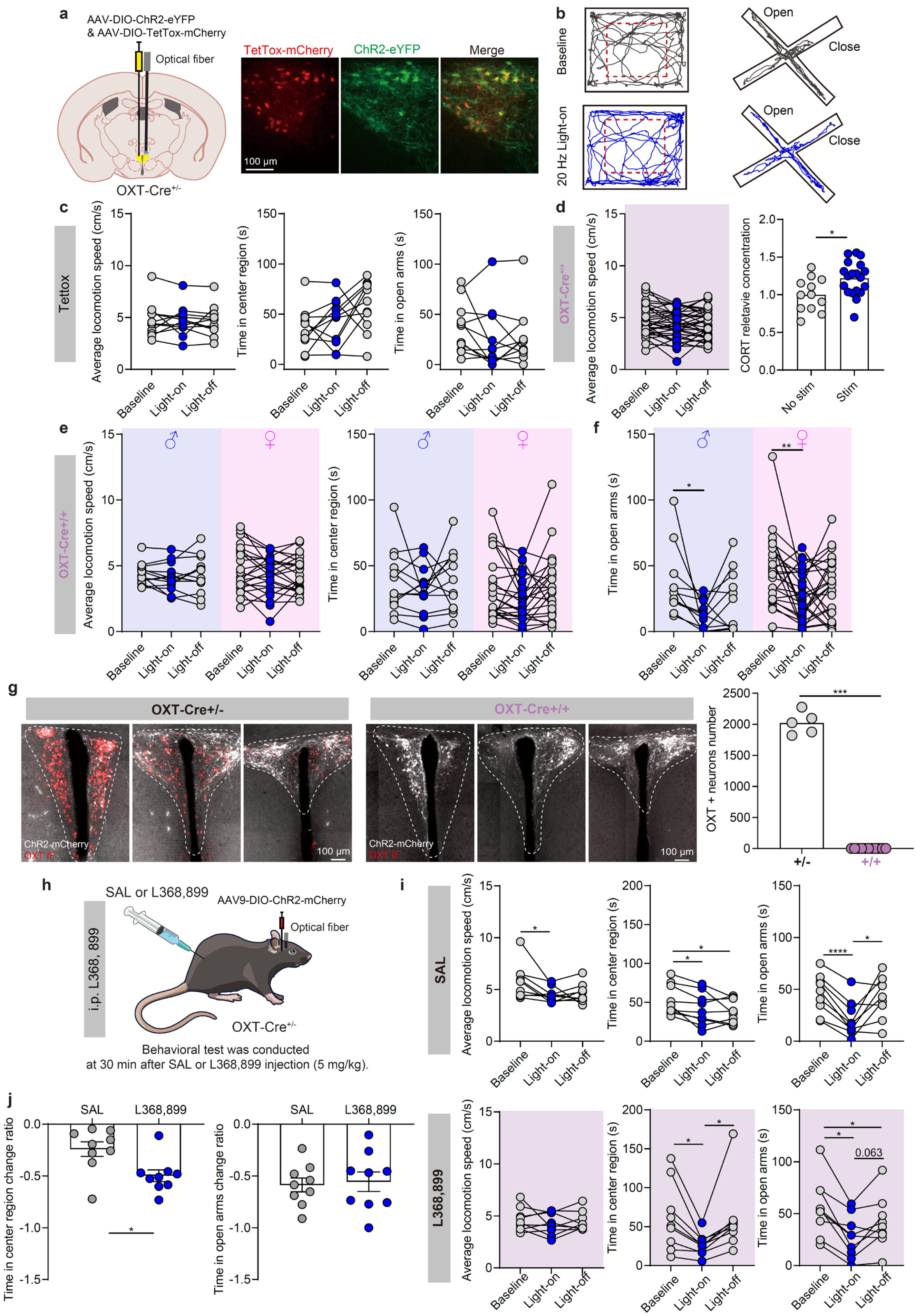

Supplementary Figure S2. Blocking synaptic vesicle release, but not OXT signaling,

abolishes the anxiogenic effect mediated by activating PVN OXT neurons. (**a**) Schematic and example of virus injection and optic fiber implantation. (**b**) Movement trajectories of example mice with both AAV-ChR2-eYFP and AAV-TetTox-mCherry virus expression in PVN OXT neurons in the OFT (Left) and EPM (Right) before and during light stimulation. (**c**) Summary of light stimulation-induced behavioral alterations in the OFT and EPM. n = 11 mice. (**d**) Summary of average locomotion speed in the OFT in OXT-Cre+/+ mice (Left). n = 36 mice. Summary of Plasma corticosterone (CORT) levels in ChR2-mCherry expression OXT-Cre+/+ mice with (Stim) and without (No stim) light stimulation. n = 12 and 9 mice for the No stim and Stim groups, respectively (Right). (**e**) Summaries about locomotion speed (Left) and time spent in the center region (Right) in males and females in the OFT. n = 13 males and 23 females. (**f**) Same as (**e**), but the EPM test. (**g**) Example and summary of ChR2-mCherry virus and OXT expression in OXT-Cre+/- and OXT-Cre+/+ mice. n = 5 OXT-Cre+/- mice and n = 11 OXT-Cre+/+ mice. (**h**) Schematic of optogenetic activation-induced behavioral alterations when intraperitoneal (i.p.) injection of saline (SAL) or oxytocin receptor antagonist L368, 899. (**i**) Summary of optogenetic activation-induced behavioral alterations in the OFT and EPM when i.p. SAL or L368, 899. n = 9 mice for SAL and L368, 899, respectively. (**j**) Summaries of the light-induced changes ratio in time spent in the center region in the OFT test and time spent in the open arms in the EPM test. n = 9 mice for SAL and L368, 899, respectively. * $p < 0.05$, ** $p < 0.01$, ***$p < 0.001$, ****$p < 0.0001$, unpaired t-test or one-way ANOVA with Tukey *post hoc* tests or Friedman test with Dunn *post hoc* tests.

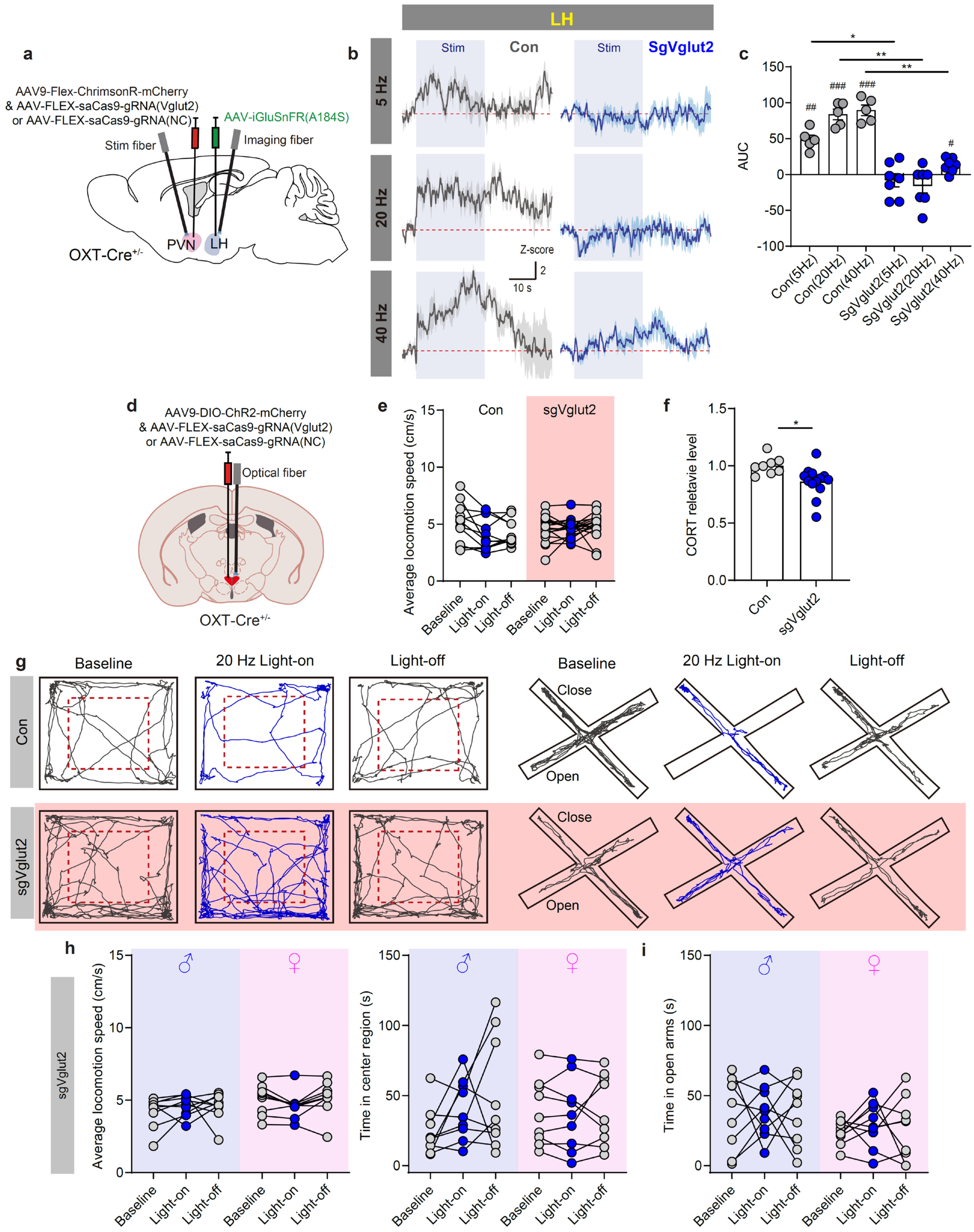

Supplementary Figure S3. Disrupting glutamate release abolishes the anxiogenic effect mediated by activating PVN OXT neurons. (**a**) Schematic of virus injection and optic fiber implantation for monitoring glutamate release in the LH while disrupting glutamate release with

activation of PVN OXT neurons. (**b**) Average changes of iGluSnFR signals in the LH of the Con and sgVglut2 groups induced by activation of PVN OXT neurons. (**c**) Summary of light stimulation-induced glutamate signal changes in the LH. n = 5 and 7 mice for Con and sgVglut2 groups, respectively. (**d**) Schematic of virus injection and optic fiber implantation to disrupt glutamate release with activation of PVN OXT neurons. (**e**) Summary of average locomotion speed in the center region in the OFT. n = 11 mice and 18 mice for the Con and sgVglut2 groups, respectively. (**f**) Summary of Plasma CORT levels in ChR2-mCherry expression Con and sgVglut2 groups with light stimulation. n = 8 and 13 mice for the Con and sgVglut2 groups, respectively. (**g**) Example trajectories in the OFT and EPM of the Con (Top) and sgVglut2 (Bottom) mice before, during, and after optogenetic activation. (**h**) Summaries about locomotion speed (Left) and time spent in the center region (Right) in males and females in the OFT when activating PVN OXT neurons after knocking down *Vglut2*. n = 8 males and 8 females. (**i**) Same as (**h**), but the EPM test. * $p < 0.05$, ** $p < 0.01$, paired *t*-test, or one-way ANOVA with Tukey *post hoc* tests, or Friedman test with Dunn *post hoc* tests, or unpaired *t*-test. ## $p < 0.01$, ### $p < 0.001$, one-sample *t*-test.

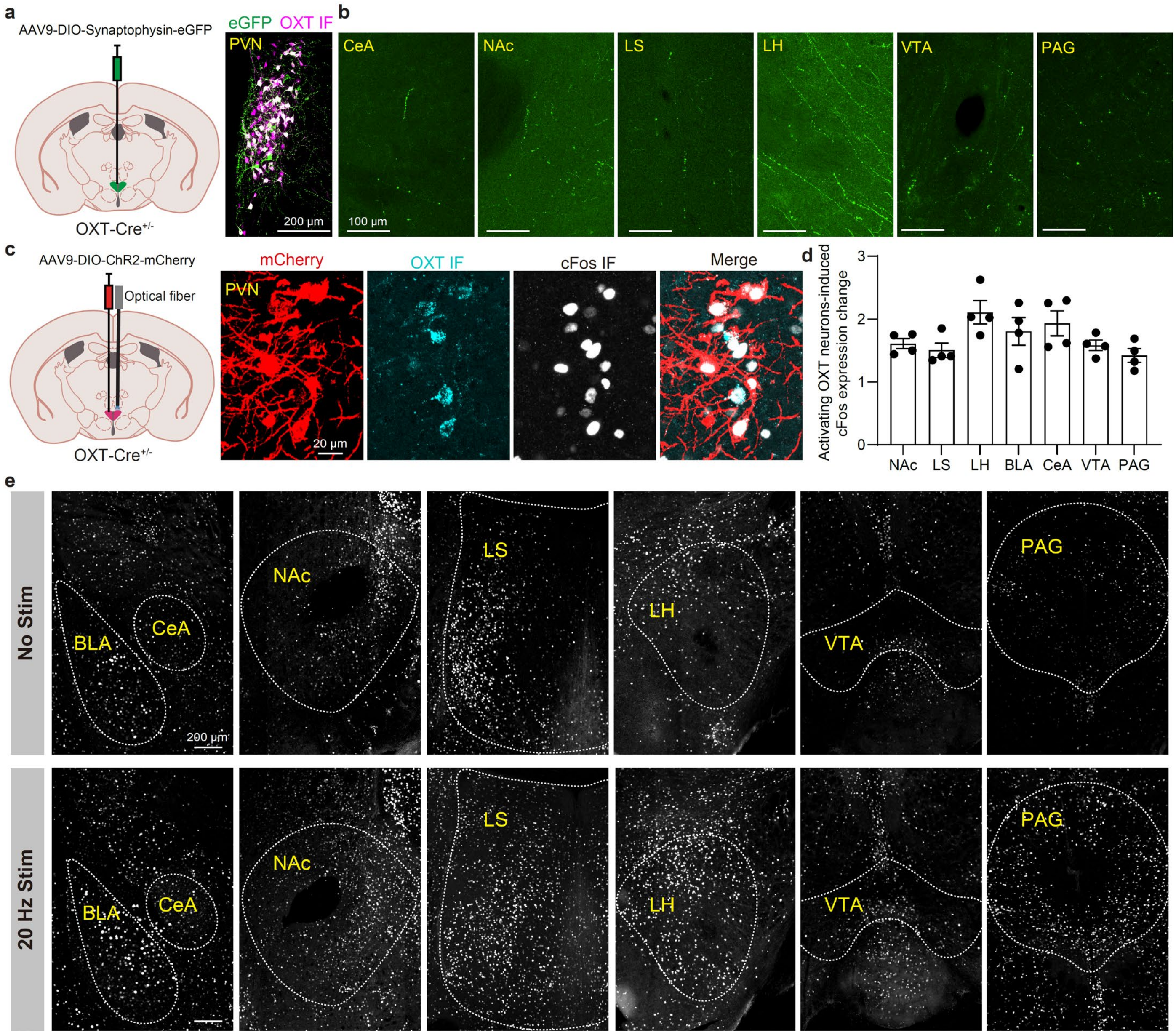

Supplementary Figure S4. The possible downstream regions of PVN OXT neurons in inducing anxiety-like behaviors. (**a**) Left: Schematic of AAV9-DIO-Synaptophysin-eGFP injection into PVN of OXT-Cre$^{+/-}$ mice. Right: Example images showing colocalization between eGFP+ neurons and OXT-immunopositive neurons in the PVN. (**b**) Example images showing eGFP+ axons distributed in the CeA, NAc, LS, LH, VTA, and PAG. (**c**) cFos expression in PVN OXT+ neurons after optogenetic activation. (**d**) Summary of cFos expression change in different brain regions after activating PVN OXT neurons. *n* = 4 mice and 4 mice for 20 Hz stim and no-stim, respectively. (**e**) Examples of cFos expression in different brain regions with (20 Hz Stim) and without (No Stim) activation of PVN OXT neurons in OXT-Cre$^{+/-}$ mice.

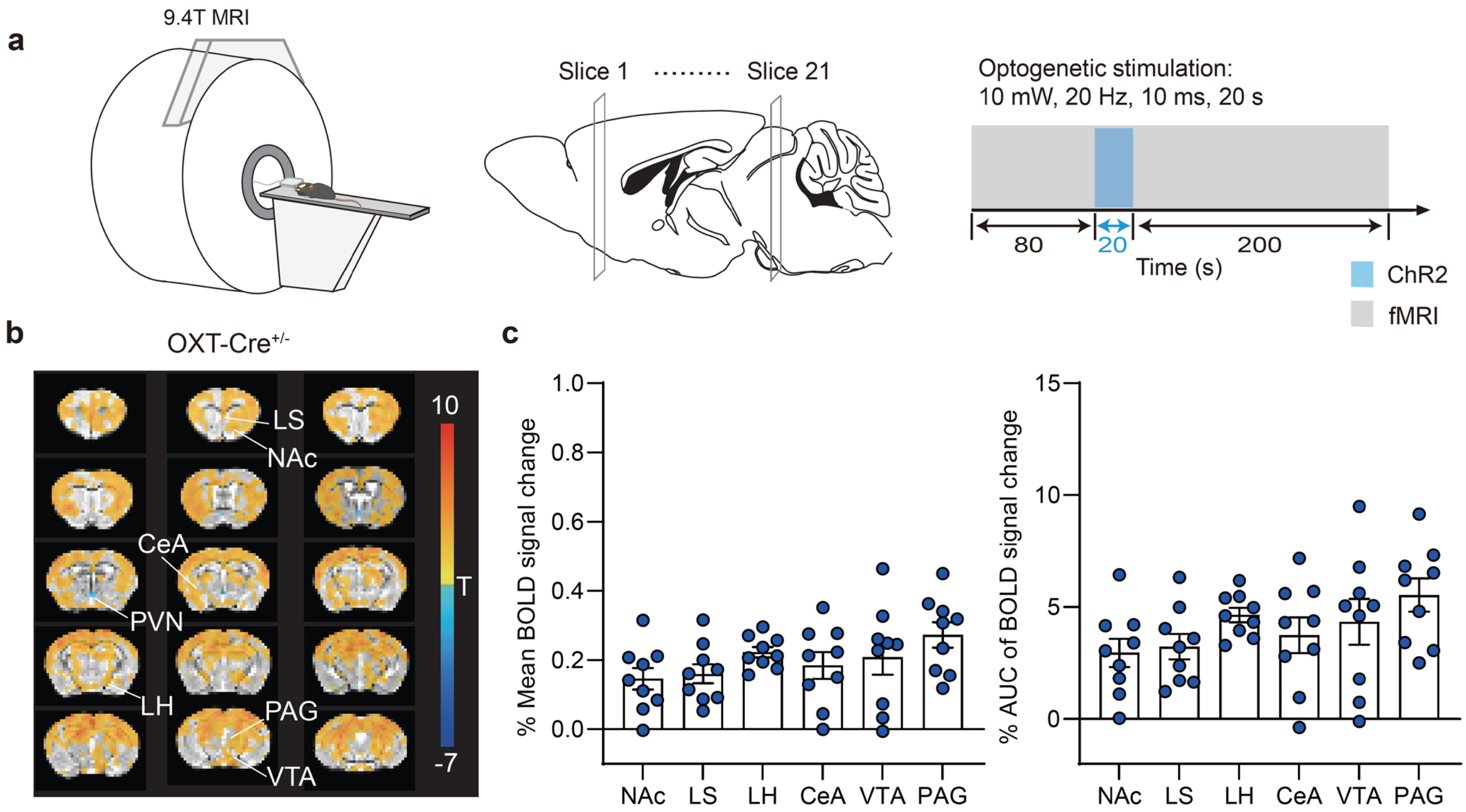

Supplementary Figure S5. Influences of optogenetic activation of PVN OXT neurons on their downstream brain regions measured by fMRI. (**a**) Schematic of optogenetic stimulation and fMRI scanning. (**b**) Statistical t-maps in OXT-Cre$^{+/-}$ mice (n = 9) with a t-threshold corresponding to p < 0.005. One-sample t-test. (**c**) Mean (Left) and AUC (Right) of over 40 s from the onset of optogenetic stimulation in NAc, LS, LH, CeA, VTA, and PAG in the OXT-Cre$^{+/-}$ group (n = 9).

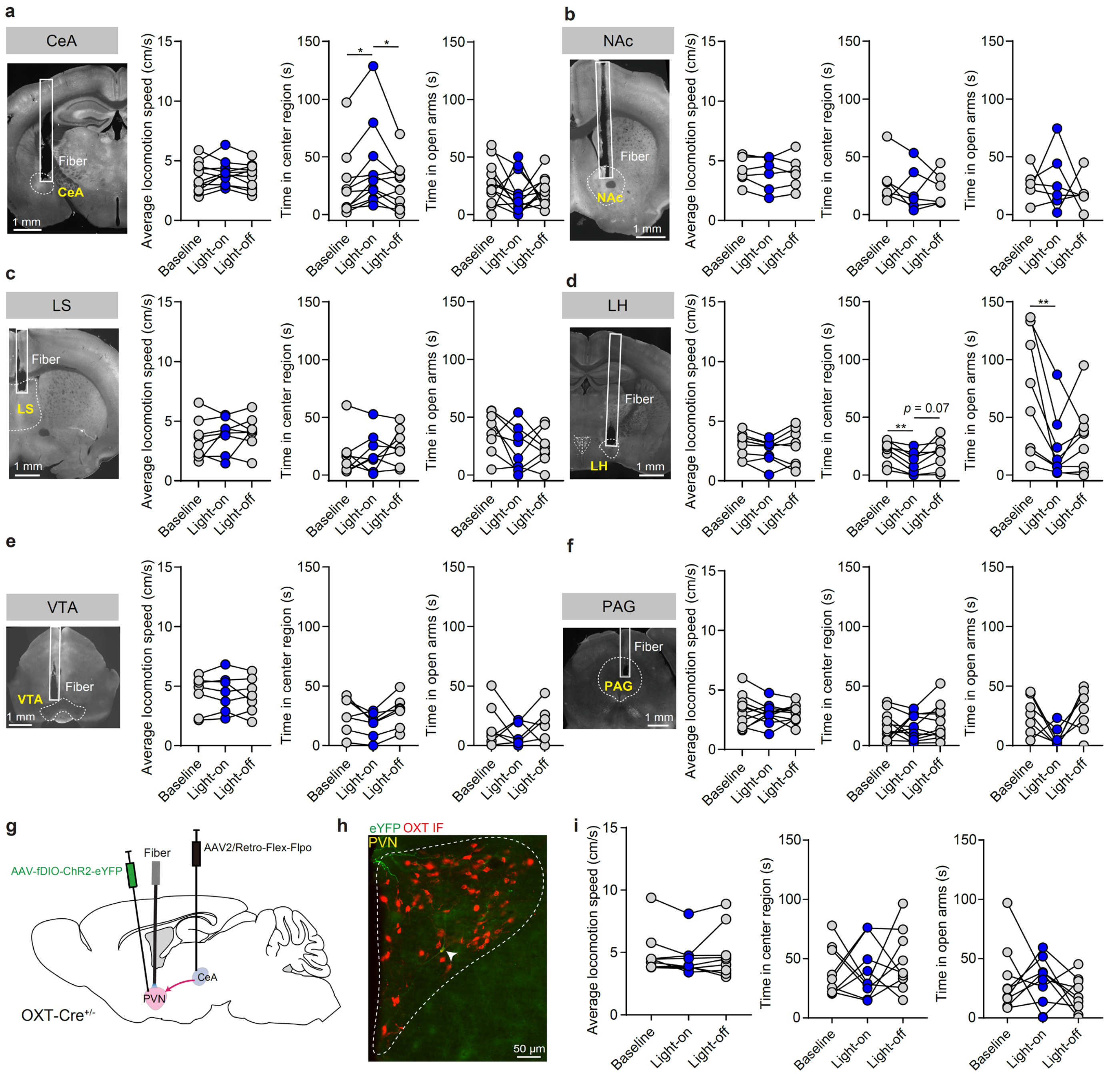

Supplementary Figure S6. Effects of activating PVN OXT neuronal projections to different downstream regions on mouse anxiety-like behaviors. (**a**) Left: An example image showing optical fiber location in the CeA. Right: Summary about average locomotion speed, time spent in the center region, and time spent in open arms in the OFT and EPM, respectively. *n* = 11 mice. (**b**) – (**f**) Same as (**a**), but for NAc (**b**), LS (**c**), LH (**d**), VTA (**e**), and PAG (**f**), respectively. *n* = 6, 8, 8, 7 mice for NAc, LS, LH, and VTA regions, respectively. *n* = 11 and 7 mice for OFT and EPM for the PAG region. (**g**) The strategy to activate CeA-projecting PVN OXT neurons. (**h**) Colocalization between eYFP+ neurons and OXT+ neurons. (**i**) Summaries about average locomotion speed, time

spent in the center region, and time spent in open arms in the OFT and EPM, when stimulating CeA -projecting PVN OXT neurons expressing ChR2-eYFP. $n = 9$ mice. * $p < 0.05$, ** $p < 0.01$, one-way ANOVA with Tukey *post hoc* tests.

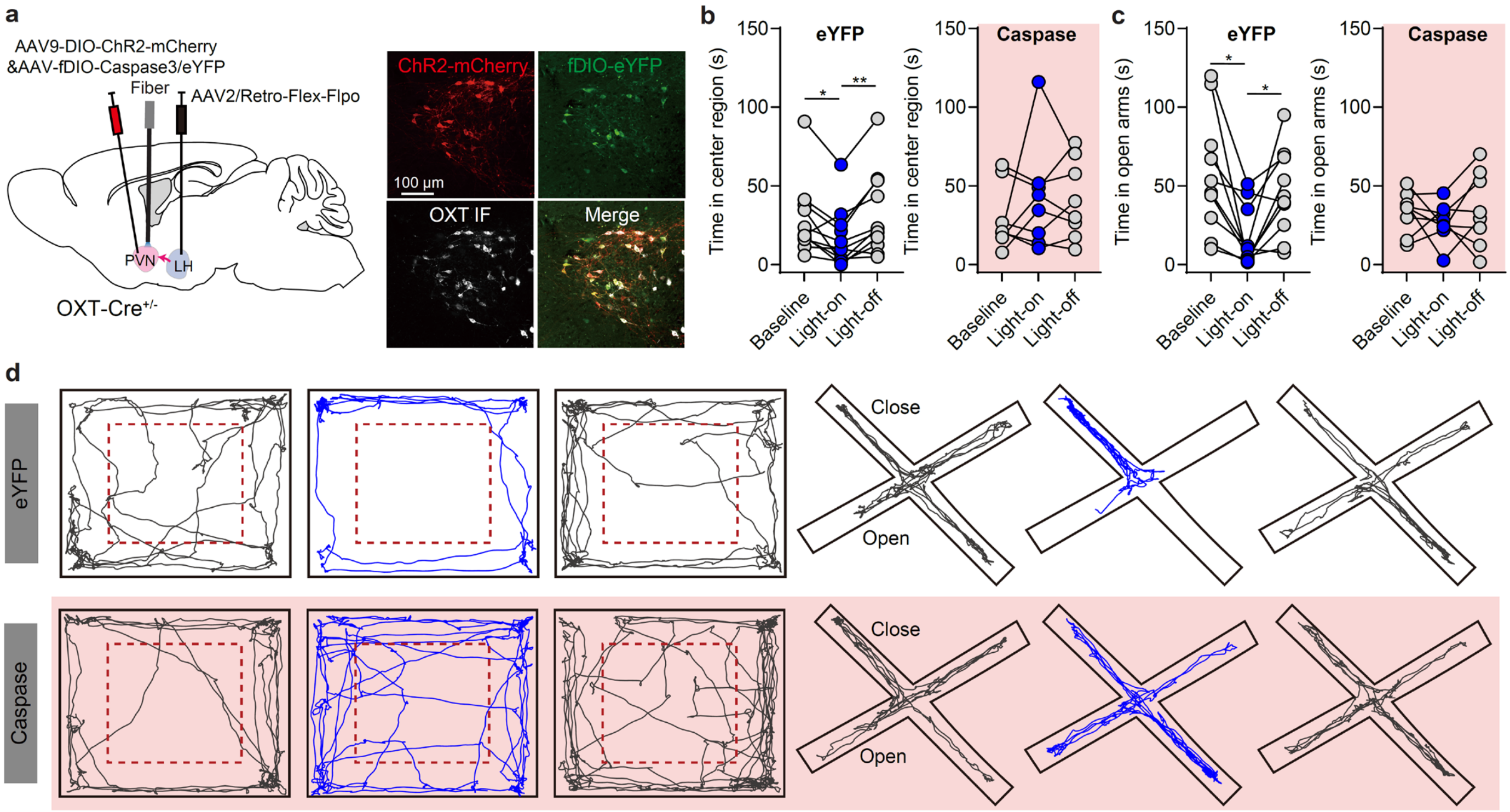

Supplementary Figure S7. PVN OXT neurons project to the LH to induce the anxiogenic effects. (**a**) Left: The strategy to activate PVN OXT neurons after ablating LH-projecting PVN OXT neurons. Right: The colocalization between ChR2-mCherry+, fDIO-eYFP+ neurons, and OXT+ neurons in the PVN. (**b**) Summary about the time spent in the center region in the OFT when activating PVN OXT neurons in the mice without (Left) and with ablating the LH-projecting PVN OXT neurons (Right). n = 11 and 8 mice for eYFP and caspase, respectively. (**c**) Same as (**b**), but for the time spent in the open arms in the EPM. (**d**) Example trajectories in the OFT and EPM of the eYFP (Top) and Caspase (Bottom) mice before, during, and after optogenetic activation. * $p < 0.05$, ** $p < 0.01$, one-way ANOVA with Tukey *post hoc* tests.

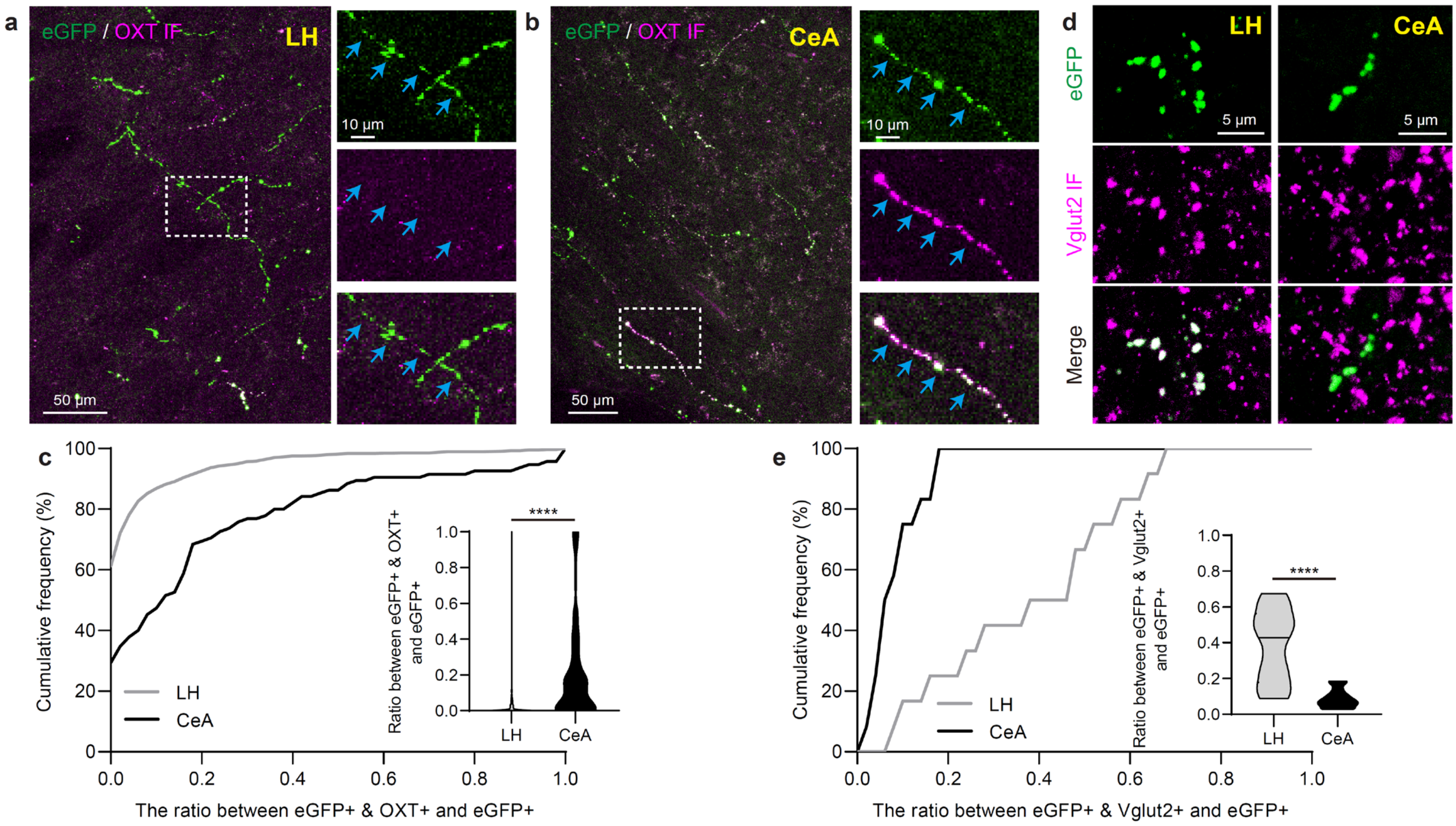

Supplementary Figure S8. PVN OXT neurons mainly release glutamate in the LH and OXT in the CeA. (**a**) Left: Example image showing the colocalization of eGFP-labeled axons (Green) from PVN OXT neurons with OXT immunofluorescence signal (Magenta) in the LH. Right: The magnified image shown in the white rectangle in the Left. (**b**) Same as (**a**), but for the CeA. (**c**) Summaries of the colocalization between eGFP-labeled axons of PVN OXT neurons and OXT immunostaining in the CeA and LH. n = 1027 and 95 axons for LH and CeA from 3 mice, **** $p < 0.0001$, Mann-Whitney test. (**d**) The example images showing the colocalization between eGFP-labeled axons and Vglut2 immunofluorescence signal in the LH (Left) and CeA (Right). (**e**) Same as (**c**), but for the colocalization between eGFP-labeled axons of PVN OXT neurons and Vglut2 immunostaining in the CeA and LH. n = 70 and 20 axons for LH and CeA from 4 mice, **** $p < 0.0001$, unpaired t-test.

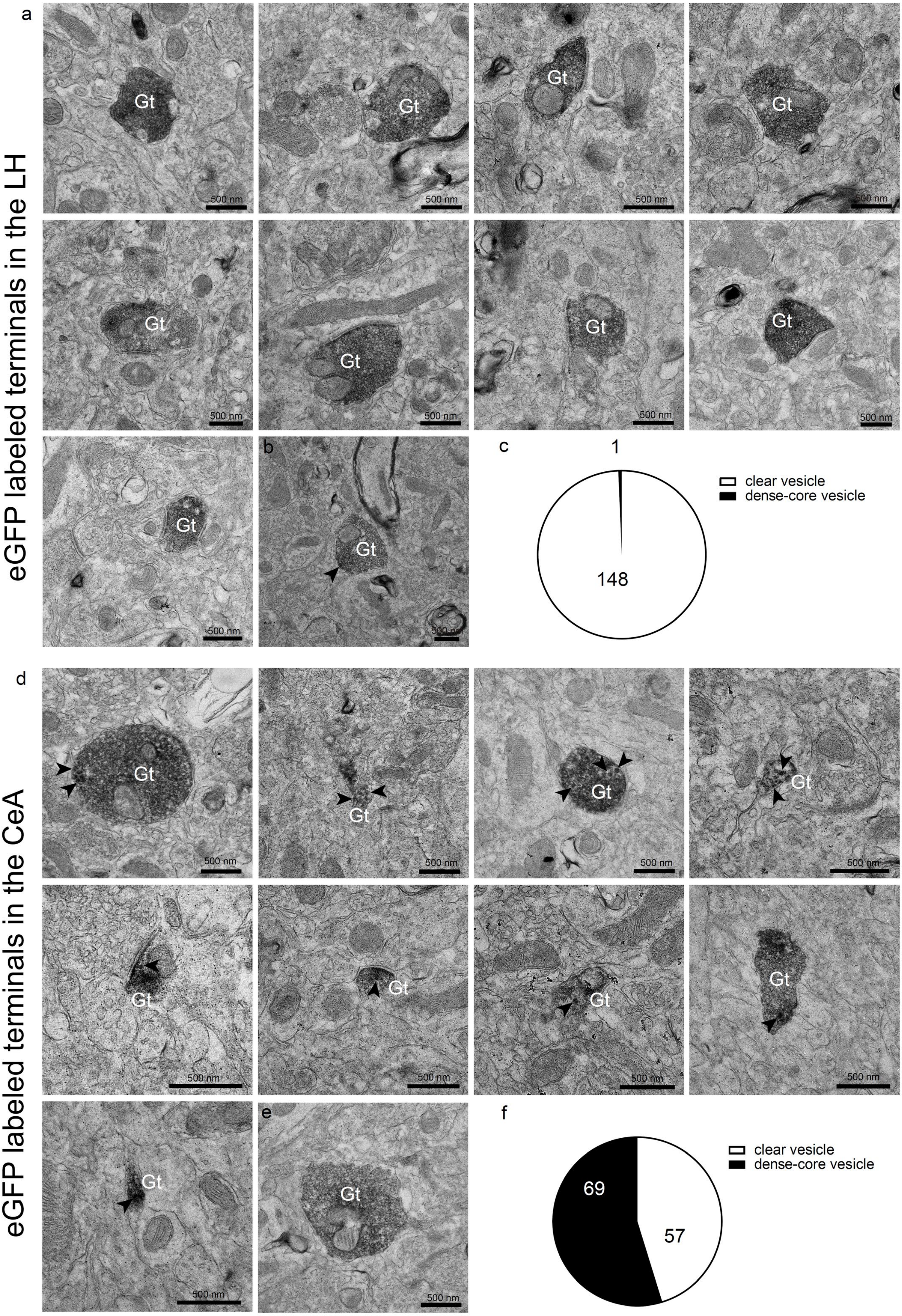
a
eGFP labeled terminals in the LH
Gt
500 nm
b
c
1
148
clear vesicle
dense-core vesicle
d
eGFP labeled terminals in the CeA
e
f
69
57

Supplementary Figure S9. The ultrastructural features of different vesicles in the eGFP terminals in the LH and CeA. (**a**) The eGFP-labeled terminals (Gt) in the LH contained small, spherical clear vesicles without large dense-core vesicles. (**b**) One eGFP-labeled terminal (Gt) in the LH contained small, spherical clear vesicles with a large dense-core vesicle. Arrowhead indicated large dense-core vesicles. (**c**) The number of eGFP-labeled terminals containing distinct types of vesicles in the LH (total 149 terminals from 2 mice). (**d**) The eGFP-labeled terminals (Gt) in the CeA contained small, spherical clear vesicles with large dense-core vesicles. Arrowhead indicated large dense-core vesicles. (**e**) One eGFP-labeled terminal (Gt) in the CeA contained small, spherical clear vesicles without large dense-core vesicles. (**f**) The number of eGFP-labeled terminals containing distinct types of vesicles in the CeA (total 126 terminals from 2 mice).

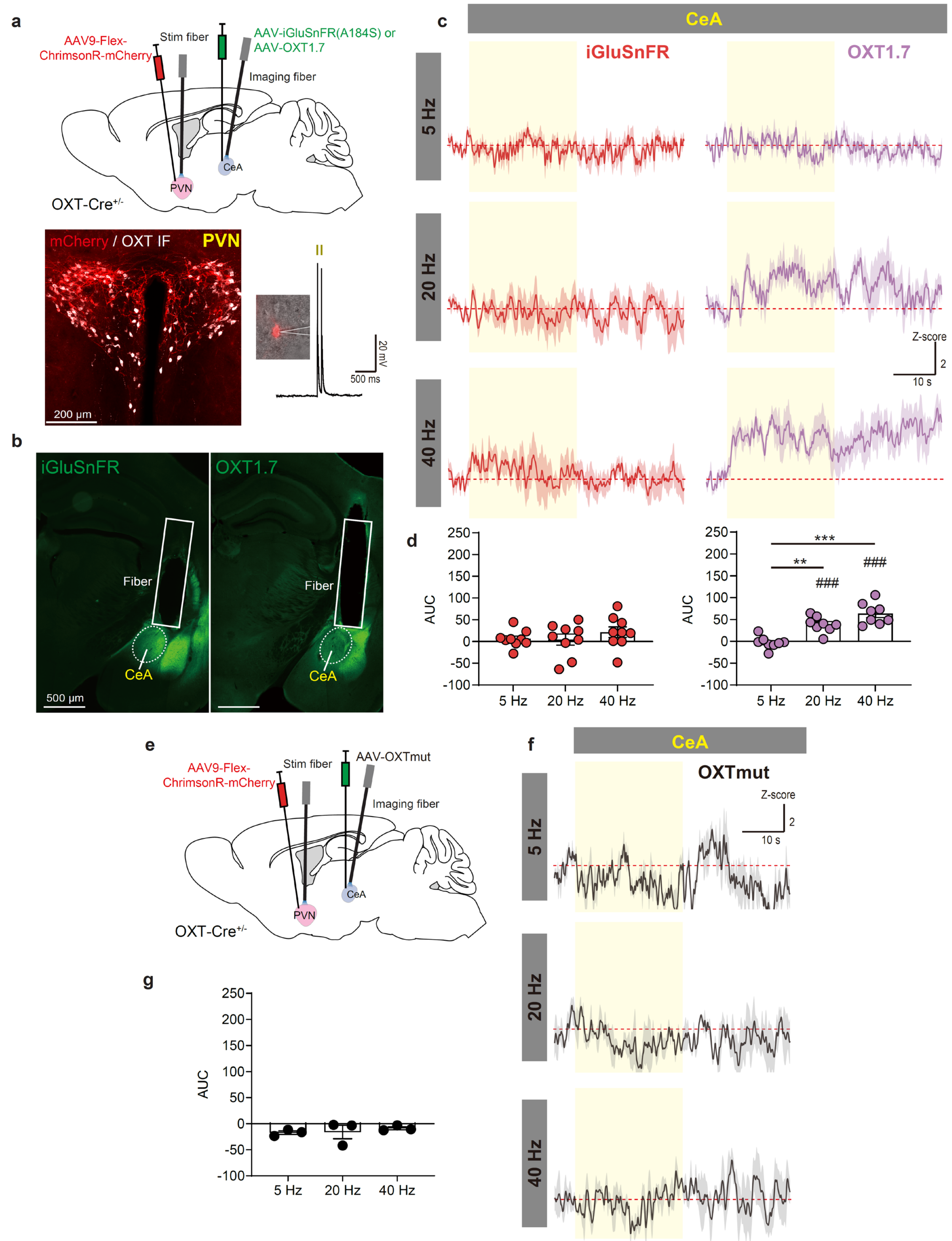

Supplementary Figure S10. PVN OXT neurons mainly release OXT in the CeA. (**a**) Top: Schematic for monitoring glutamate and OXT release in the CeA upon optogenetic stimulation of PVN OXT neurons. Bottom: AAV-Flex-ChrimsonR-mCherry expression in PVN OXT neurons and

example responses of one mCherry+ neuron evoked by 647 nm red light. (**b**) Example images showing iGluSnFR expression, OXT1.7 expression, and fiber implantation in the CeA. (**c**) Average changes of iGluSnFR (Left) and OXT1.7 (Right) signals in the CeA induced by activation of PVN OXT neurons at 5Hz, 20Hz, and 40Hz. (**d**) Summary of light stimulation-induced glutamate (Left) and OXT (Right) signal changes in the CeA. n = 9 and 8 mice for iGluSnFR and OXT 1.7 groups, respectively. (**e**) Schematic for monitoring OXT release with mutant OXT probe in the CeA. (**f**) Average changes of OXTmut signals in the CeA induced by activation of PVN OXT neurons at 5Hz, 20Hz, and 40Hz. (**g**) Summary of light stimulation-induced OXT signal changes in the CeA with mutant OXT probe. n = 3 mice. ### $p < 0.001$, one-sample t-test; ** $p < 0.01$, *** $p < 0.001$, one-way ANOVA with *post hoc* Tukey's multiple comparisons test.

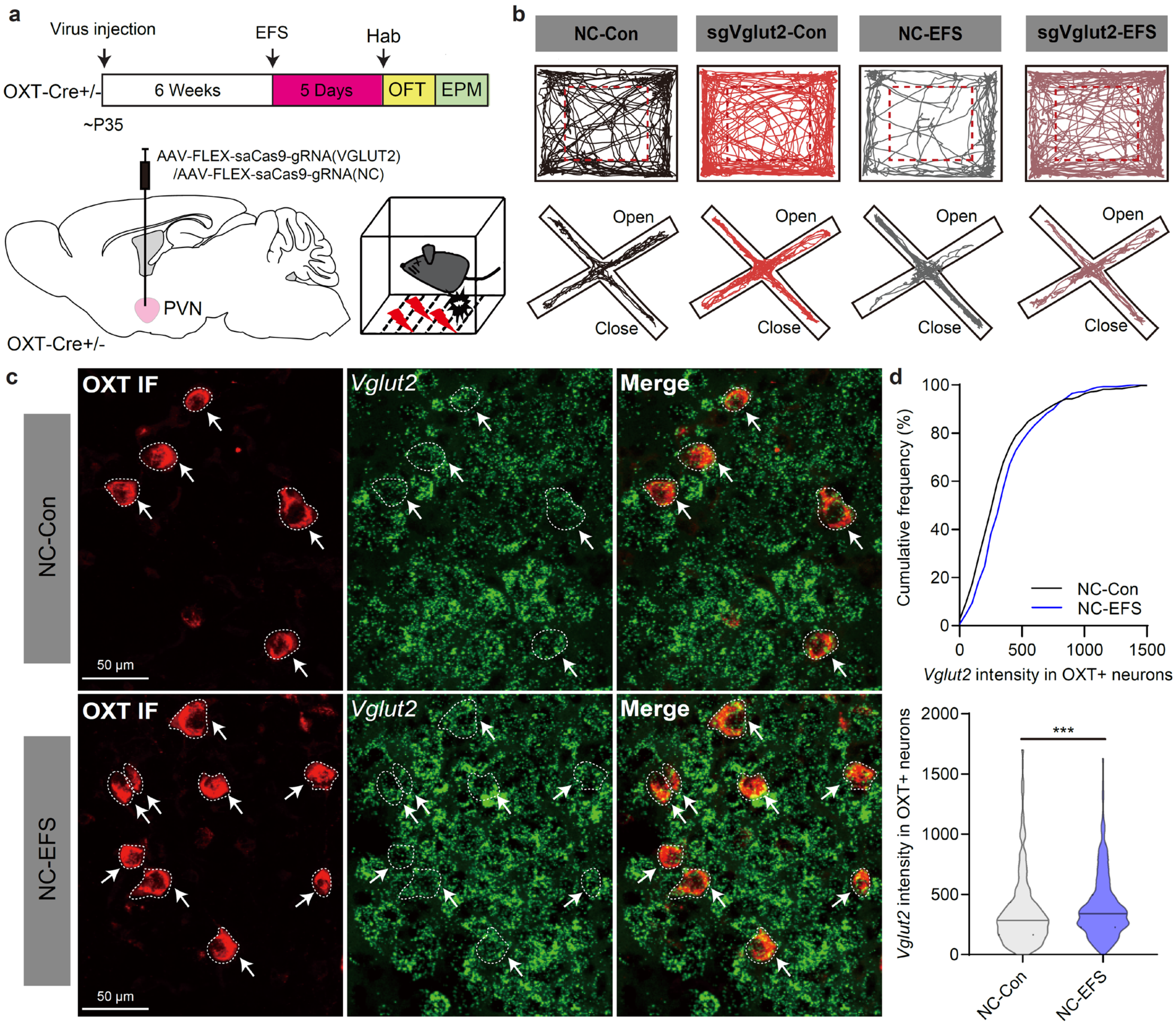

Supplementary Figure S11. Disrupting glutamate release from PVN OXT neurons rescues the electrical foot stimulation (EFS)-induced anxiety-like behaviors. (**a**) Top: Experimental protocol. Bottom: Virus injection strategy and the electrical foot stimulation (EFS) diagram. (**b**) Top: Example trajectories of the NC-Con, sgVglut2-Con, NC-EFS, and sgVglut2-EFS mice in the OFT. The red dashed rectangles indicate the center region. Bottom: Example trajectories of the NC-Con, sgVglut2-Con, NC-EFS, and sgVglut2-EFS mice in the EPM. (**c**) Example of EFS-induced *Vglut2* change in PVN OXT neurons. (**d**) Summary of EFS-induced *Vglut2* change in PVN OXT neurons. n = 385 and 401 OXT neurons from 4 NC-Con mice and 3 NC-EFS mice, *** $p < 0.001$, Mann-Whitney test.

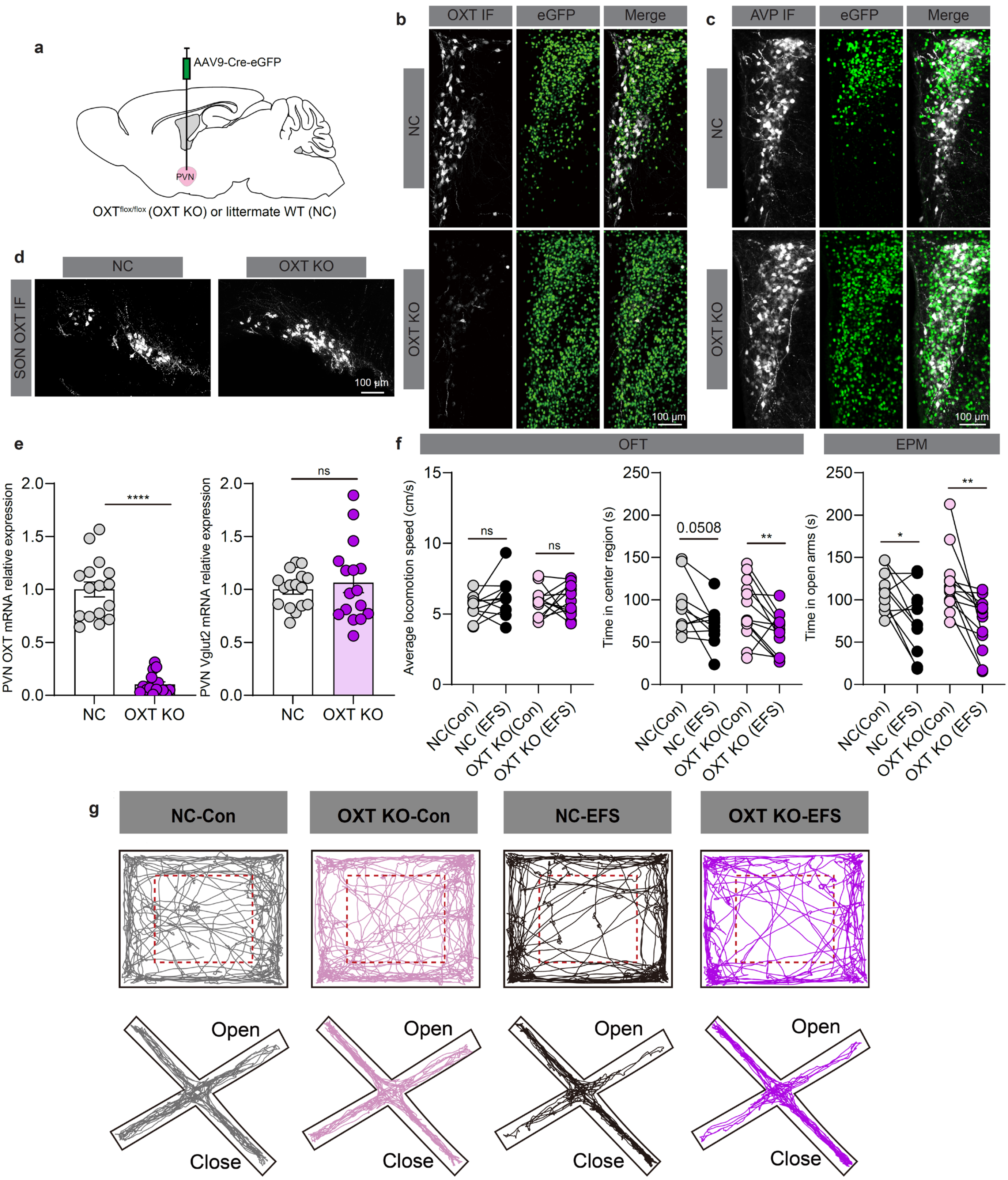

Supplementary Figure S12. Knockdown of OXT expression in PVN OXT neurons did not change the electrical foot stimulation (EFS)-induced anxiety-like behaviors. (**a**) Virus injection strategy. (**b**) Example images showing OXT immunostaining and eGFP virus expression in the PVN in littermate WT (NC) and OXT knockdown (OXT KO) mice. (**c**) Example images showing arginine

vasopressin (AVP) immunostaining and eGFP virus expression in the PVN in littermate WT (NC) and OXT knockdown (OXT KO) mice. (**d**) Example images showing OXT immunostaining and eGFP virus expression in the SON in littermate WT (NC) and OXT knockdown (OXT KO) mice. (**e**) PVN OXT mRNA (Left) and Vglut2 mRNA (Right) relative level in NC and OXT KO groups. $n = 16$ mice and 16 mice for NC and OXT KO groups, respectively. (**f**) Summary of average locomotion speed and time spent in the center region in the OFT, and time spent in the open arms and the number entering the open arms in the EPM for NC and OXT KO mice subjected to the EFS. $n = 11$ mice and 13 mice for NC and OXT KO groups, respectively. (**g**) Top: Example trajectories of the NC-Con, OXT KO-Con, NC-EFS, and OXT KO-EFS mice in the OFT. The red dashed rectangles indicate the center region. Bottom: Example trajectories of the NC-Con, OXT KO-Con, NC-EFS, and OXT KO-EFS mice in the EPM. * $p < 0.05$, ** $p < 0.01$, **** $p < 0.0001$, unpaired t-test or paired t-test.
